# Functional and evolutionary characterization of potential auxiliary metabolic genes of the global RNA virome

**DOI:** 10.1002/imo2.70002

**Published:** 2025-02-11

**Authors:** Yang Zhao, Zhihao Zhang, Meiling Feng, Rong Wen, Pengfei Liu

**Affiliations:** ^1^ College of Ecology Lanzhou University Lanzhou China; ^2^ Center for Pan‐Third Pole Environment Lanzhou University Lanzhou China; ^3^ Key Laboratory of Pan‐Third Pole Biogeochemical Cycling Lanzhou China; ^4^ Chayu Monsoon Corridor Observation and Research Station for Multi‐Sphere Changes Chayu China; ^5^ State Key Laboratory of Tibetan Plateau Earth System, Resources and Environment (TPESRE), Institute of Tibetan Plateau Research Chinese Academy of Sciences Beijing China; ^6^ University of Chinese Academy of Sciences Beijing China

## Abstract

We generated the first comprehensive view of RNA viral auxiliary metabolic genes (AMGs), expanding the known functional type of AMGs by 75%. RNA viruses encode a remarkably high diversity of AMGs, spanning 25 distinct functional categories. Most of these genes are linked to environmental regulation and genetic information processing, with fewer associated with nutrient cycling. Additionally, RNA viruses carrying AMGs are capable of infecting both eukaryotes and prokaryotes, and may acquire AMGs from organisms beyond their predicted host range.

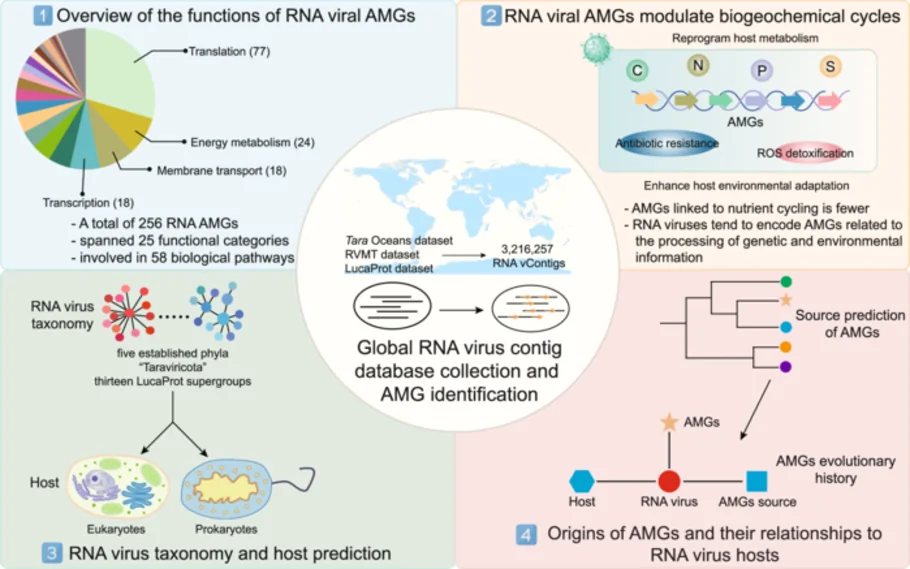

Viruses are the most abundant biological entities on Earth, exhibiting immense diversity and playing vital ecological roles [1]. They can influence crucial ecological processes and biogeochemical cycles by altering host metabolism via auxiliary metabolic genes (AMGs) [2]. AMGs are virus‐encoded genes acquired from the host and sporadically present in the phage genome to a relatively unknown degree [3]. Viruses sample host genes during infection, and a subset of these horizontal gene transfer events will be kept in the viral genome if they augment or redirect important metabolic processes that can provide sufficient adaptive advantages to viruses under specific environmental conditions [4].

For decades, research on AMGs has focused on DNA viruses [4]. DNA viral genomes from a variety of habitats—including humans, oceans, soils, wastewater treatment plants, and extreme environments [5]—have revealed diverse AMGs with functions ranging from photosynthesis, carbon and phosphate metabolism, nitrogen and sulfur cycling, nucleic acid metabolism, antioxidants, and heavy metal detoxification [4, 6].

The development of metatranscriptomic sequencing has illuminated the vast, untapped diversity of global RNA viruses, revealing their equally critical ecological roles in shaping host communities, driving evolution, and regulating host metabolism [7]. RNA viral AMGs, a key mechanism of these effects, remain largely unexplored, with only a few studies focusing on specific environments [8, 9, 10, 11, 12]. The global diversity and ecological significance of RNA viral AMGs, however, remain poorly understood. By leveraging the power of newly available global RNA virome datasets—including the *Tara* Oceans data set [10], the RNA Viruses in Metatranscriptomes (RVMT) database [13], and the LucaProt resource [14]—this study provides a functional and phylogenetic analysis of RNA viral AMGs. By addressing this critical gap in viral ecology, we aim to uncover the ecological functions, virus–host interactions, and evolutionary dynamics of RNA viral AMGs on a global scale.

## 1 RESULTS AND DISCUSSION

### Taxonomy and hosts of RNA viruses with AMGs

**FIGURE 1:**
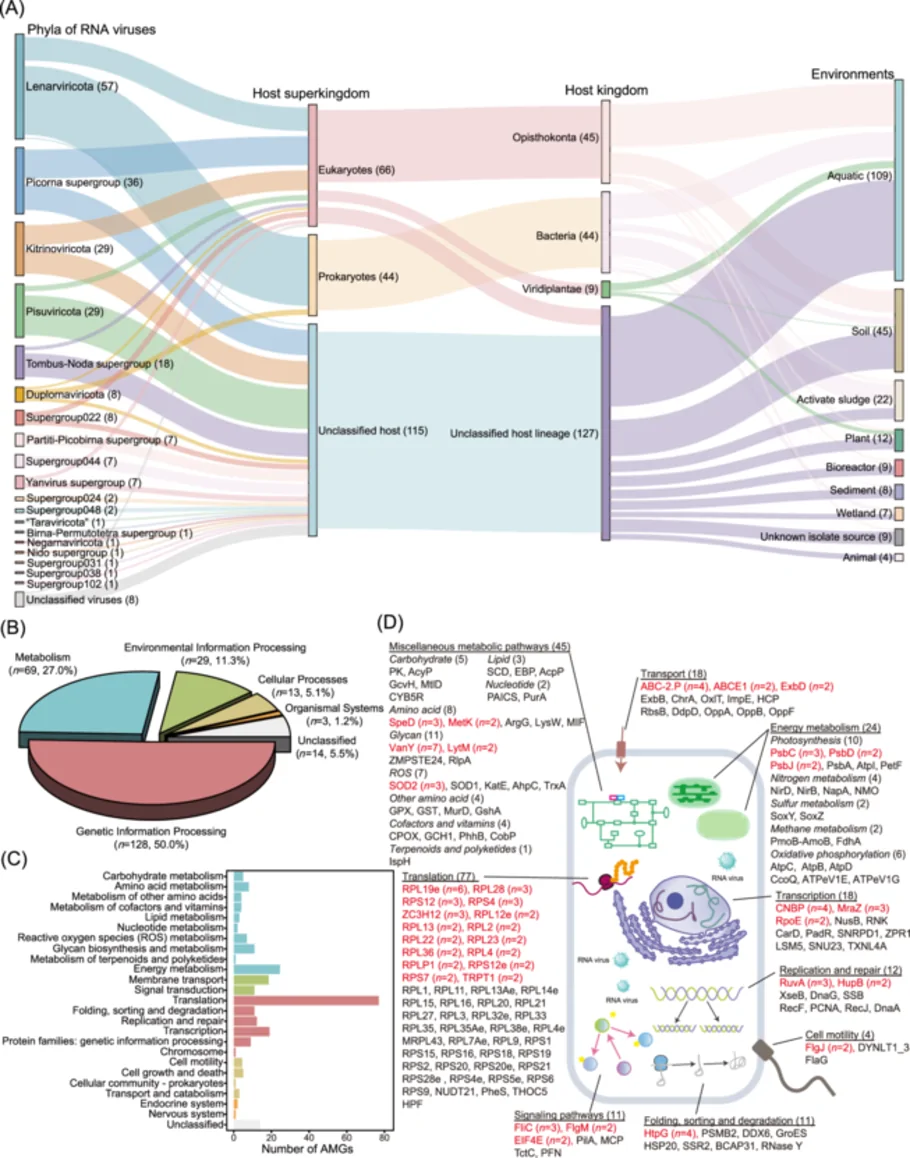
Predicted RNA virus–host interactions and overview of the RNA virus AMGs functions. (A) Linkages of RNA vContigs encoding AMGs, their infected hosts, and the environments in which they are detected. The phylum‐level taxonomy, predicted hosts and source habitats are shown on the left, the middle two columns and the right columns, respectively. (B) The percentage of viral AMGs involved in metabolism, environmental information processing, genetic information processing, cellular processes, and organismal systems. (C) Distribution of 256 RNA viral AMGs in 25 function categories. (D) Number of AMGs associated with main metabolic function categories.

By screening 3,216,257 RNA viral genomes (vContigs) from global RNA virome datasets, we identified 256 putative AMGs from 225 RNA vContigs (Figure S1, Table S1). Of these RNA viruses, 124 were assigned to five established phyla: Lenarviricota (57, 25.3%), Pisuviricota (29, 12.9%), Kitrinoviricota (29, 12.9%), Duplornaviricota (8, 3.6%), and Negarnaviricota (1, 0.4%). Additionally, 93 vContigs were classified under the novel “Taraviricota” (1, 0.4%) and 13 distinct LucaProt supergroups (92, 40.9%) (Figure 1A). The LucaProt supergroups primarily consisted of Picorna (36, 16.0%), Tombus‐Noda (18, 8.0%), and Supergroup022 (8, 3.6%) (Figure 1A). However, 8 RNA viruses (3.6%) remained unclassified. Among the taxonomically assigned RNA viruses, Fiersviridae (34, 15.1%), Narnaviridae (12, 5.3%), and Marnaviridae (12, 5.3%) were the most represented families (Figure S2, Table S2).

Host predictions revealed that RNA viruses with AMGs predominantly infect eukaryotes (66, 29.3%), including Opisthokonta (Fungi and Metazoa) and Viridiplantae (green plants). Among these, Opisthokonta emerged as the most frequent eukaryotic host group for these RNA viruses (Figure 1A, Table S2). Meanwhile, 44 RNA viruses (19.6%) with AMGs belonged to canonical prokaryotic RNA viruses, including Leviviricetes and Vidaverviricetes [13]. The hosts of the remaining 115 RNA viruses (51.1%) could not be confidently assigned.

### Overview of RNA viral AMG functions

The 256 AMGs were involved in 58 biological pathways (Figure S3), categorized into metabolic (69, 27.0%), genetic (128, 50.0%), and environmental (29, 11.3%) information processing, as well as cellular processes (13, 5.1%) and organismal systems (3, 1.2%) (Figure 1B). These pathways spanned 25 functional categories, with the top four being translation (77, 30.1%), energy metabolism (24, 9.4%), membrane transport (18, 7.0%), and transcription (18, 7.0%) (Figure 1C). The most abundant category was ribosomal proteins (RPs) (Figure 1D), which are critical for ribosome assembly and protein synthesis, as viruses rely entirely on the host's translational machinery. After infection, viruses can selectively inhibit the synthesis of host protein by usurping endogenous translation pathways and increasing the biosynthesis of RNA virus proteins [15]. The high prevalence of RPs in RNA viruses highlights their importance in viral replication.

Interestingly, we found that 18 RNA vContigs encoded more than one AMG (Figure S4). Notable examples include Pisuviricota vContigs 150DCM1MMQQ14_25606 and 152SUR2MMQQ14_439061, which encoded photosynthesis‐related proteins PsbC and PsbD; Lenarviricota vContig S11_len2823, which encoded sulfur metabolism proteins SoxY and SoxZ; and Lenarviricota vContig ND_008319, which encoded oxidative phosphorylation proteins AtpC and AtpD, all of which are central to energy metabolism (Figure S4). Furthermore, a vContig from Kitrinoviricota (TARA_131_DCM_0.22‐3_k119_171980) encoded enzymes involved in nitrogen metabolism (NapA, NirB, and NirD), alongside the ribosomal protein RPS21, and the DNA priming enzyme DnaG, suggesting regulation of nitrogen metabolism, protein synthesis, and DNA replication.

### RNA viral AMGs modulate host environment adaptation

RNA viral AMGs can encode numerous chaperone proteins or peripheral proteins to regulate host environment sensing and stress adaptation (Figure 1D). For example, the molecular chaperone (HtpG) and ATP‐dependent Clp protease (ClpP) encoded by AMGs may increase host cell tolerance to high temperatures [16]. AMGs involved in flagellar formation can enhance the ability of host cells to adhere, invade, and acquire nutrients by avoiding hostile environments [17]. RNA vContigs can also remodel the host antioxidant network and improve viral replication efficiency by encoding several oxidoreductases (catalase, superoxide dismutase, peroxiredoxin, and thioredoxin) for scavenging reactive oxygen species (Figure 1D).

Given the close evolutionary relationship between viruses and their hosts, RNA viral AMGs may reflect adaptations to specific environmental conditions. For example, RNA viruses living in the plant phyllosphere have type VI secretion system (T6SS)‐related AMGs (HCP and ImpE) (Table S1). RNA viruses carrying these AMGs can help plant hosts improve their environmental adaptations by inhibiting plant pathogens and responding to abiotic stresses [18]. Meanwhile, AMGs encoding coproporphyrinogen III oxidase (CpoX), a key enzyme in tetrapyrrole synthesis, were identified in RNA viruses from plant rhizosphere (Table S1). RNA viruses with the *cpoX* gene may prevent light‐dependent cell death of the plant host and promote its growth [19]. RNA viruses living in mammals were enriched with AMGs encoding the oligopeptide transport system (Table S1), which can mediate the uptake of dipeptides and tripeptides by the animal host and provide it with a nitrogen source [20]. In environments such as soil and activated sludge, RNA viral AMGs encoding antibiotic resistance proteins (Table S1), like VanY, may reflect adaptation to high concentrations of antibiotics in these habitats.

### RNA viral AMGs modulate biogeochemical cycles

**FIGURE 2:**
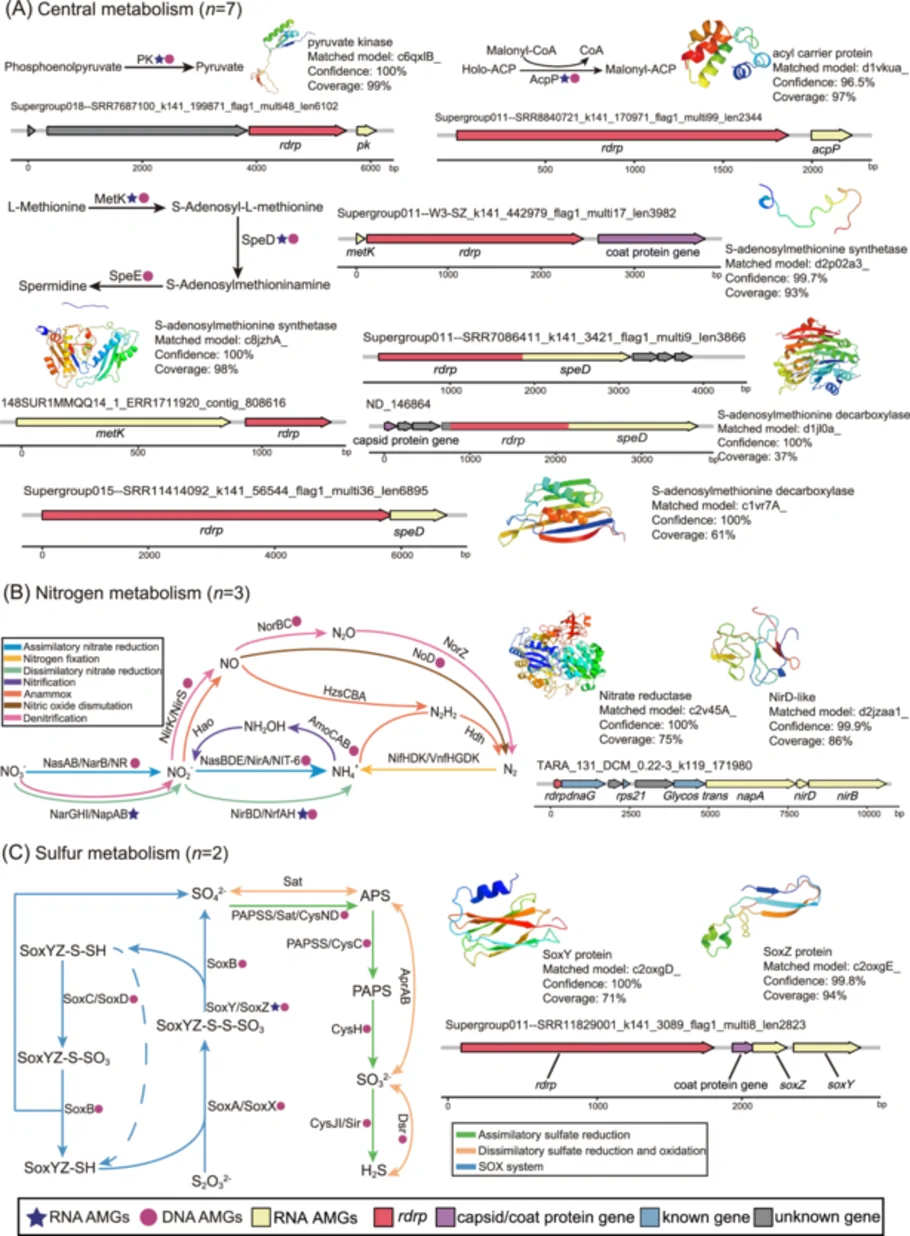
The AMGs encoded by RNA vContigs revealed the involvement of RNA viruses in central, nitrogen, and sulfur metabolism. (A) Metabolic pathways with *pk*, *acpP*, *speD*, and *metK* gene, the reference protein model for the PK, AcpP, SpeD, and MetK, and the genome architecture for the RNA vContig encoding central metabolism genes. Supergroup011‐‐SRR7086411_k141_3421_flag1_multi9_len3866 and ND_146864 share the same SpeD protein structure model. (B) Potential contribution of AMGs to nitrogen metabolism, genome architecture of RNA vContig encoding nitrogen metabolism genes and reference protein models for NapA and NirD. (C) Potential contribution of AMGs to the sulfur cycling, reference protein models for SoxY and SoxZ, and the genome architecture of RNA vContig encoding sulfur cycling associated genes. Panels (A)–(C) share the legend. DNA AMGs indicate the counterpart of AMGs was also found in DNA viruses.

To further investigate how RNA viruses may impact biogeochemical cycles, 13 representative AMGs were selected for analysis. These genes are associated with central metabolism (pyruvate kinase, *pk*; acyl carrier protein, *acpP*; S‐adenosylmethionine synthesis, *metK*; and S‐adenosylmethionine decarboxylase, *speD*), nitrogen metabolism (nitrate reductase, *napA*; nitrite reductase large subunit, *nirB*; and nitrite reductase small subunit, *nirD*), sulfur metabolism (sulfur‐oxidizing proteins, *soxY* and *soxZ*), and photosynthesis systems (photosystem II (PSII) D1 protein, *psbA*; PSII D2 protein, *psbD*; PSII CP43 reaction center protein, *psbC*; and PSII reaction center protein J, *psbJ*) (Figure 2, Figure S5). The protein structure modeling showed that the confidence of these proteins as PK, AcpP, MetK, SpeD, NapA, NirD, SoxY, SoxZ, PsbA, PsbC, PsbD, and PsbJ were all more than 98% (Figure 2, Figure S5). Although NirB was not confirmed by protein modeling, its full amino acid sequence shares 100% identity with the NCBI reference sequence for NirB (Accession No.: WP_105027197.1) (Table S3).

DNA viruses encode numerous AMGs related to carbon, nitrogen, phosphorus, and sulfur metabolism [4, 6]. In contrast, RNA viruses have only a few of AMGs involved in nitrogen and sulfur metabolism, and none related to phosphorus cycling, suggesting that RNA viruses may have a more restricted role in nutrient cycling than their DNA counterparts. The host range differences between RNA and DNA viruses might contribute to this difference, as RNA viruses predominantly infect eukaryotes, while DNA viruses mainly target prokaryotes, including bacteria and archaea.

### Comparison of the predicted host of RNA viruses and the potential origins of AMGs

To investigate the relationship between the potential sources of AMGs and the hosts of RNA viruses encoding them, we constructed phylogenetic trees for 30 types of AMGs (62 sequences from 51 RNA vContigs) associated with biogeochemical cycling, protein synthesis, and motility. Phylogenetic analysis revealed that 36 AMGs encoded by 27 RNA vContigs originated from prokaryotes, while the predicted hosts for 8 vContigs encoding 11 AMG sequences were eukaryotes (Figures S6, S7A–K and Table S4). Of the remaining 19 RNA vContigs, six were predicted to infect unknown prokaryotes (Figures S6, S7L–P and Table S4), and 13 infected hosts of unknown identities (Figure S6, Table S4).

Phylogenetic analysis also indicated that 26 AMGs from 24 RNA vContigs were of eukaryotic origin, with two vContigs (S11_len3866 and ND_146864) encoding SpeD predicted to infect prokaryotes (Figures S6, S7A). Among the remaining 22 RNA vContigs, 10 were predicted to infect eukaryotes and 12 infected hosts with unknown identities (Figures S6, S8A–H and Table S4). Therefore, 10 (19.6%) of these investigated RNA vContigs had a host that was inconsistent with the source of their encoded AMGs (Table S4).

The potential hosts of these RNA viruses are primarily assigned according to their taxonomic classification. However, the prediction of uncultured RNA viruses is still a challenging task. Recent studies revealed expanding lineages of prokaryotes RNA viruses [9]. For example, the subsets of picobirnaviruses and partitiviruses known as eukaryote RNA viruses, were most likely to be able to infect prokaryotes [13]. Therefore, we propose that these eukaryote RNA viruses encoding prokaryotes originated AMGs might represent novel lineages of prokaryote RNA viruses.

Alternatively, it could also be that these RNA viruses and bacteria have the same eukaryote hosts. During the co‐infection, RNA viruses, bacteria and eukaryote hosts form a tripartite association and RNA viruses of eukaryotes might obtain bacteria encoded genes. In addition, it could be that the RNA virus AMGs is acquired by their hosts through HGT events from a third organism. Finally, we acknowledge that some RNA viral contigs may result from misassembles. While the RNA viral contigs in this study were assembled *de novo* from short reads and the risk of chimeric assemblies is low, it cannot be entirely excluded. Future studies employing long‐read sequencing or efforts to isolate these RNA viruses will be essential for resolving these uncertainties and shedding light on the ecological and evolutionary roles of RNA viral AMGs in host‐virus interactions.

### Limitation

During the preparation of this work, a few studies on RNA virus AMGs were published as preprints, which are not included in the current paper [9, 12]. Compared with these studies, similar patterns were observed for the AMGs encoded by RNA viruses from these environments, which are primarily associated with transcription, translation, signaling pathways, membrane transport and photosynthesis. The widespread distribution of these AMGs emphasizes their importance in regulating the metabolism and virus–host interactions of RNA viruses in these ecosystems. Compared to the published datasets of RNA viral AMGs [9, 10, 11, 12], 75.3% (140 out of 186 different types of AMGs) of the AMGs identified in this analysis are new (Table S5), which significantly expands the existing diversity of global RNA viral AMGs. Currently, only a small portion of environments of the global ecosystems are covered by RNA virome studies. Together with our synthesis, we predict that a greater diversity of RNA viral AMGs will be uncovered with a more comprehensive sampling of the global RNA virome.

## 2 CONCLUSION

Leveraging global RNA virome datasets, we generated the first comprehensive view of RNA viral AMGs. Our findings revealed several key insights: (i) RNA viruses exhibit remarkably high AMG diversity, spanning 25 distinct functional categories; (ii) AMGs predominantly encode proteins involved in the regulation of environmental and genetic information processing, while those associated with nutrient cycling are less common; (iii) the hosts of RNA viruses carrying AMGs include both eukaryotes and prokaryotes; and (iv) RNA viruses may acquire AMGs from organisms outside their predicted host range. Collectively, these findings significantly enhance our understanding of RNA viral ecology, shedding light on the intricate roles RNA viruses play in modulating host metabolism and ecosystem functions.

## 3 METHODS

Detailed procedures for data collection, identification of AMGs, functional annotation analysis, and phylogenetic tree construction are comprehensively described in the Supporting Information Methods, including related Figures S9–10 and Table S6.

## AUTHOR CONTRIBUTIONS

**Yang Zhao**: Data curation; software; investigation; validation; visualization; writing—original draft; writing—review and editing; methodology. **Zhihao Zhang**: Methodology; software; investigation; writing—review and editing. **Meiling Feng**: Writing—review and editing; investigation. **Rong Wen**: Writing—review and editing; investigation. **Pengfei Liu**: Conceptualization; methodology; writing—review and editing; writing—original draft; investigation; funding acquisition; supervision.

## CONFLICT OF INTEREST STATEMENT

The authors declare no conflicts of interest.

## ETHICS STATEMENT

No animals or humans were involved in this study.

## Supporting information


**Figure S1.** Overview of the global RNA viral AMGs analysis pipeline.
**Figure S2.** Phylogenetic tree of RdRps for four phyla.
**Figure S3.** RNA viral AMGs are distributed in eight habitats and the 58 biological pathways.
**Figure S4.** Network diagram of 18 RNA vContigs encoding multiple AMGs.
**Figure S5.** The AMGs encoded by RNA vContigs revealed the involvement of RNA viruses in photosynthesis system.
**Figure S6.** Network diagram of RNA vContigs with host information and their AMGs sources, including AMGs related to biogeochemical cycling, protein synthesis, and motility.
**Figure S7.** Phylogenetic tree of RNA AMGs that may have originated in prokaryotes.
**Figure S8.** Phylogenetic tree of RNA AMGs that may have originated in eukaryotes.
**Figure S9.** Genome architecture of RNA vContig encoding a ribosomal protein gene and reference protein model.
**Figure S10.** RdRP sequence with three highly complete conserved sequence motifs (A, B, and C).

**Table S1.** Auxiliary metabolism genes (AMGs) detected in the global RNA virus dataset.
**Table S2.** RNA viruses with AMGs and host linkage.
**Table S3.** Protein model predictions for AMGs.
**Table S4.** The relationship between the potential sources of AMGs and the hosts of RNA viruses.
**Table S5.** 140 new RNA AMGs found in this study and 46 previously reported RNA viral AMGs.
**Table S6.** HHsuite annotation results for RNA vContigs with AMGs.

## Data Availability

The data that support the findings of this study are available from the corresponding author upon reasonable request. The data and scripts used in this study can be found at https://github.com/YangZhao-LZU/RNA_AMG. Supplementary materials (methods, figures, tables, graphical abstract, slides, videos, Chinese translated version, and update materials) may be found in the online DOI or iMeta Science http://www.imeta.science/imetaomics/.
