# Supplementary material for: Functional and evolutionary characterization of potential auxiliary metabolic genes of the global RNA virome
Source: IMetaOmics. 2025 Feb 11;2(2):e70002. doi: 10.1002/imo2.70002 (PMC12806420; doi:10.1002/imo2.70002)
Supplement: Supplementary file 1 — Figure S1. Overview of the global RNA viral AMGs analysis pipeline. Figure S2. Phylogenetic tree of RdRps for four phyla. Figure S3. RNA viral AMGs are distributed in eight habitats and the 58 biological pathways. Figure S4. Network diagram of 18 RNA vContigs encoding multiple AMGs. Figure S5. The AMGs encoded by RNA vContigs revealed the involvement of RNA viruses in photosynthesis system. Figure S6. Network diagram of RNA vContigs with host information and their AMGs sources, including AMGs related to biogeochemical cycling, protein synthesis, and motility. Figure S7. Phylogenetic tree of RNA AMGs that may have originated in prokaryotes. Figure S8. Phylogenetic tree of RNA AMGs that may have originated in eukaryotes. Figure S9. Genome architecture of RNA vContig encoding a ribosomal protein gene and reference protein model. Figure S10. RdRP sequence with three highly complete conserved sequence motifs (A, B, and C). [file IMO2-2-e70002-s001.docx]

**Supporting information to：**

**Functional and evolutionary characterization of potential auxiliary metabolic genes of the global RNA virome**

**Running title:** Functional diversity of global RNA viral AMGs

Yang Zhao^1,2,3,4^, Zhihao Zhang^5,6^, Meiling Feng^1,2,3,4^, Rong Wen^1,2,3,4^, Pengfei Liu^2,3,4^*

^1^College of Ecology, Lanzhou University, Lanzhou, 730000, China

^2^Center for Pan-third Pole Environment, Lanzhou University, Lanzhou, 730000, China

^3^Key Laboratory of Pan-third Pole Biogeochemical Cycling, Lanzhou, 730000, China

^4^Chayu Monsoon Corridor Observation and Research Station for Multi-Sphere Changes, Chayu, 860600, China

^5^State Key Laboratory of Tibetan Plateau Earth System, Resources and Environment (TPESRE), Institute of Tibetan Plateau Research, Chinese Academy of Sciences, Beijing, 100101, China

^6^University of Chinese Academy of Sciences, Beijing, 100101, China

*Correspondence: [liupf@lzu.edu.cn](mailto:liupf@lzu.edu.cn) (Pengfei Liu)

**METHODS**

## Global RNA virome genome data collection

We compiled a global RNA viral contig (vContigs) dataset comprising 3,216,257 RNA vContigs from three sources: the *Tara* Oceans RNA viral dataset (TO), The RNA Viruses in Metatranscriptomes (RVMT) database, and the LucaProt RNA virome dataset, all previously published [1–3]. The TO dataset represents the first global marine RNA virus genome catalog, derived from 771 marine metatranscriptomes (187 prokaryote-enriched and 584 eukaryote-enriched) sampled from 121 oceanic stations during the *Tara* Oceans and *Tara* Oceans Polar Circle expeditions. A total of 44,779 RNA vContigs were identified from these metatranscriptomes. The RVMT dataset encompasses a broader environmental scope, covering aquatic, marine, freshwater, soil, plant, animal, and engineered systems, and includes 5150 metatranscriptomes, from which 2,658,344 RNA vContigs were identified. Finally, the LucaProt dataset covered 10,487 metatranscriptomes from diverse ecosystems, identifying 513,134 RNA vContigs through a deep-learning-based analysis using the LucaProt algorithm.

## Identification of the auxiliary metabolic genes (AMGs)

To date, there is no dedicated bioinformatic tool tailored to annotate putative auxiliary metabolic genes (AMGs) in RNA viruses. However, Dominguez-Huerta et al. suggested that ‘cellular’ protein sequences identified in RNA virus genomes may parallel the AMGs that are ecologically important in DNA viruses [2]. Therefore, we identified the putative AMGs encoded by RNA vContigs through a combination of functional annotation and manual curation. Initially, all 3,216,257 RNA vContigs were annotated using the Distilled and Refined Annotation of Metabolism (DRAM v1.4.6) pipeline [4]. DRAM is a tool for genome annotation, and KOfam, UniRef-90, PFAM-A, dbCAN, RefSeq viral, VOGDB, and the MEROPS peptidase databases were used for homolog search and virus genome annotations in the current study. Following DRAM annotation, we manually filtered the results to focus only on genes involved in cell-associated metabolic processes, which were classified as putative AMGs. To verify the ‘cellular’ origin, each putative AMG was blast against the NCBI nr database (Release May, 2024), and only those with top match (bit score > 50) that were cellular were kept [2]. For AMGs with inconsistent annotations according to different databases, we applied HHsuit to annotate RNA vContigs carrying AMGs and Phyre2 for structure annotation of these AMGs to further validate their functionality (Figure S9, Table S3, Table S6) [5,6]. Examples of putative AMGs include genes encoding enzymes for metabolism and enhancement of virus and host survivability (e.g., biosynthesis and carbohydrate metabolism). In addition, genes encoding conserved RNA viral proteins, including viral invasion (i.e., glycoside hydrolases and peptidases involved in cell wall lysis), modification of viral components (i.e., glycosyl transferases, adenylyltransferases, and methyltransferases that putatively involved in viral DNA, RNA, and structural proteins modification), viral structural proteins (e.g., viral coat/core proteins and pilin-like proteins), proteins for viral replication, and assembly (e.g., DNA and RNA polymerases, and nucleotide binding proteins), were excluded [7,8].

## Novelty assessment of AMGs

Since the sequences of identified AMGs were not provided in these studies [2,9–12], we did not carry out sequence similarity-based comparisons. Instead, we collected the information of AMGs in the supplemental tables of these articles, and compared the KO terms or enzymes description of AMGs between this work and these published AMGs [2,9–12]. If the KO terms or enzymes presented in our dataset but not in the published list, they were considered as novel RNA viral AMGs.

## Identification and validation of RNA viruses with AMGs

The CDS prediction for all Contigs were carried out by using Prodigal v2.6.3 [13] with the ‘-p meta’ option setting and default genetic codon table. The obtained protein sequence was used for RNA-dependent RNA polymerase (RdRp) validation. First, the RdRp of RNA viruses were screened using LucaProt with default parameters and a threshold of 0.5 [3]. Second, protein sequences encoded by all putative RNA vContigs with AMGs were subjected to the Palmscan algorithm [14] screening based on PALMdb. Sequences with a palmprint (a segment of the palm sub-domain robustly delineated by well-conserved catalytic motifs) score ≥ 20 were regarded as positive RdRp sequences. The presence of key RdRp motifs–motif A [DxxxxD], motif B [(S/T)Gxxx(T/G)xxxN], and motif C [(S/G/N/A)D(D/N)]–were examined for all identified RdRps through multiple sequence alignments (Figure S10). Finally, the RdRp sequences were further checked by comparison with the NCBI non-redundant (nr) protein database (Release May, 2024) using blastp with an e-value threshold of 1E-3. Although the complete A-B-C motifs were not found in 20 Contigs (Table S2), we did not exclude them because these Contigs had been identified as RNA viruses by rigorous methods in previous studies [1,3,15], and these sequences were also identified as RdRp of RNA viruses by comparison with the NCBI nr database.

## Taxonomy and host prediction of RNA viruses with AMGs

In this study, we used RNA virus species and host information from Dominguez-Huerta et al., Zayed et al., Neri et al., and Hou et al., which were primarily predicted based on RdRp sequence similarity and RNA virus taxonomy [1–3,15]. The TO dataset identified 5504 “species”-level viral operational taxonomic units (vOTUs) belonging to 10 phyla, including five established and five new proposed phyla (“Taraviricota”, “Pomiviricota”, “Arctiviricota”, “Paraxenoviricota”, and “Wamoviricota”) [2]. These RNA viruses are known to infect various ecologically important organisms, primarily protists and fungi, with fewer infecting bacteria and invertebrate metazoans [2]. In the RVMT dataset, RNA viral diversity expanded from 13,282 to 124,873 distinct clusters at a taxonomic level between species and genus. Additionally, two candidate phyla (p.0001.base-Kitrino and p.0002.base-Kitrino) were proposed alongside the five previously established phyla. This dataset revealed that most RNA viruses are associated with eukaryotic hosts, with only two groups, leviviruses (Leviviricetes) and cystoviruses (Vidaverviricetes), known to infect bacteria [1]. The LucaProt dataset identified 161,979 putative viral species and 180 RNA viral supergroups, many of which are comparable to known virus phyla and classes defined by the International Committee on Taxonomy of Viruses (ICTV). Of these supergroups, only 21 corresponded to existing ICTV-classified viral phyla/classes, while 60 of the remaining 159 represented highly divergent, previously unrecognized supergroups [3].

To further infer the putative host of RNA viruses, we used five approaches (Table S2):

i) Taxonomy based. The known RNA virus taxa (phyla, classes, and families) were used to retrieve information of putative hosts according to ICTV meta information.

ii) iPHoP based. iPHoP v1.3.3 with default parameter to create link between RNA virus with prokaryotes through CRISPR spacer sequences [16].

iii) EVE based. EVEs are nucleotide sequences derived from DNA or RNA viruses that have integrated into host genomes. For this third approach, all nucleotide sequences from cellular organisms available in NCBI GenBank release 243 were used as a nucleotide database. To avoid including exogenous RNA virus genomes in the database, we excluded sequences shorter than 45 kb since the longest RNA virus genome reported so far is 45 kb [3]. To assess the evolutionary relationship of vContigs to EVEs, the near-complete RdRp protein sequences were searched against the nucleotide database by using tblastn algorithm. As previously described [17], the thresholds were set to 100 amino acids for alignment length and 1 × 10^-20^ for e-value.

iv) Hallmark gene based. RNA viruses with AMGs were subjected to functional domain annotation using three methods (see “domain and functional annotation of RNA viral genomes with AMGs”) and searched for plant (movement protein) and bacteria (i.e., lytic protein, phage coat, phage maturation, and phage integrase) related marker genes within them [1].

v) Ribosomal binding site (RBS) motif based. RBS identification were carried out as described in Schulz et al [18]. Briefly, Prodigal was run as described above (see “identification and validation of RNA viruses with AMGs”), we then retrieved the “rbs_motif” field from Prodigal’s GFF output files and classified the different 5’-UTR sequences as either “Shine Dalgarno (SD)”, “None” and “Other”. The following motifs were considered as SD-like: 3Base_5BMM, 4Base_6BMM, AGG, AGGA, AGGA_GGAG_GAGG, AGGAG, AGGAG_GGAGG, AGGAG(G)_GGAGG, AGGAGG, AGxAG, AGxAGG_AGGxGG, GAG, GAGG, GAGGA, GGA, GGA_GAG_AGG, GGAG, GGAG_GAGG, GGAGG, GGAGGA, and GGxGG [1].

## Domain and functional annotation of RNA viral genomes with AMGs

The 225 RNA vContigs with AMGs were further subjected for domain annotation by using three methods: (i) Open reading frames (ORFs) based on default code. The nucleotide sequences of 225 vContigs were annotated by InterProScan v5.62-94.0 [19] against PRINTS v.42.0 [20], Phobius v.1.0.1 [21], MobiDBLite v.2.0 [22], and TMHMM v.2.0c [23] database. (ii) ORFs based on optimized code. The optimization code was determined by the longest sequence translated by transeq (EMBOSS v.6.5.7.0) and its corresponding code table. For RNA viral genome domain annotation, ORFs were identified by using Prodigal v2.6.3 with the optimized genetic code. Profiles of amino acid sequences were generated using HHsuite v3.3.0 [6] and UniRef30_2020_03 [24] database with one iteration of hhblits under the option ‘-n 1 -e 0.001’. Then, Pfam 35 database was used to identify structural domains in the amino acid sequences and the domains annotated using hits with > 95% probability score [15]. (iii) Domain-based. Since there is still a challenge to determine the use of the code, we also performed functional domain annotation on the translated RNA vContigs sequences. Protein sequences from contigs translated by transeq for each RNA vConitgs were annotated by hmmsearch [25], which matched these proteins to Hidden Markov Models (HMM) collected from multiple protein mapping databases (CDD v.3.19 [26], Pfam 35 [27], Gene3D v4.3 [28], LysDB [1], ECOD 2017 release [29]), with a maximum e-value of 0.001. The genome architecture of RNA vContigs with AMGs was visualized using gggene (<https://github.com/wilkox/gggenes>).

## Phylogenetic analysis of RdRp and AMGs

We constructed phylogenetic trees of RdRp amino acid (AA) sequences from the four RNA virus phyla with the highest number of AMGs, including Lenarviricota, the Picorna supergroup, Pisuviricota, and Kitrinoviricota. Reference sequences were obtained from the TO, RVMT and LucaProt datasets. Briefly, reference RdRps and RdRps of RNA viruses with AMGs that from the same phylum were pooled together and aligned using MUSCLE5 with default parameters [30]. The alignment of sequences were filtered using trimAl v1.5.0 (-gappyout parameter) [31] and a custom script (fasta_drop.py) to remove sequences with > 70% gaps. The final multiple sequence alignment was used for maximum likelihood tree construction using MEGA software with poisson model [32,33]. The tree topology was evaluated using the bootstrap analysis based on 1,000 resampling replicates.

To investigate the probable evolutionary origin of the AMGs, homolog sequences of AMGs were recruited from the NCBI nr database (Release May, 2024), and the top 20 hits with a bit score > 50 were retained as reference sequences for the AMGs. The AMGs and reference sequences were aligned using MUSCLE5, and manually checked and trimmed as described above for RdRps. The phylogenetic trees were constructed using the methods described above and visualized using the iTOL online server [34]. We regard the sources of gene sequences (e.g., prokaryotes or eukaryotes) that cluster closely with AMG in the phylogenetic tree as potential sources of AMG.

**REFERENCES**

1. Neri, Uri, Yuri I. Wolf, Simon Roux, Antonio Pedro Camargo, Benjami Lee, Darius Kazlauskas, I. Min Chen, et al. 2022. “Expansion of the global RNA virome reveals diverse clades of bacteriophages.” *Cell* 185: 4023-4037. <https://doi.org/10.1016/j.cell.2022.08.023>

2. Dominguez-Huerta, Guillermo, Ahmed A. Zayed, James M. Wainaina, Jiarong Guo, Funing Tian, Akbar Adjie Pratama, Benjamin Bolduc, et al. 2022. “Diversity and ecological footprint of global ocean RNA viruses.” *Science* 376: 1202-1208. <https://doi.org/10.1126/science.abn6358>

3. Hou, Xin, Yong He, Pan Fang, Shi-Qiang Mei, Zan Xu, Wei-Chen Wu, Jun-Hua Tian, et al. 2024. “Using artificial intelligence to document the hidden RNA virosphere.” *Cell* 187: 1-14. <https://doi.org/10.1016/j.cell.2024.09.027>

4. Shaffer, Michael, Mikayla A. Borton, Bridget B. McGivern, Ahmed A. Zayed, Sabina Leanti La Rosa, Lindsey M. Solden, Pengfei Liu, et al. 2020. “DRAM for distilling microbial metabolism to automate the curation of microbiome function.” *Nucleic Acids Research* 48: 8883-8900. <https://doi.org/10.1093/nar/gkaa621>

5. Kelley, Lawrence A., Stefans Mezulis, Christopher M. Yates, Mark N. Wass, Michael J. E. Sternberg. 2015. “The Phyre2 web portal for protein modeling, prediction and analysis.” *Nature Protocols* 10: 845-858. <https://doi.org/10.1038/nprot.2015.053>

6. Steinegger, Martin, Markus Meier, Milot Mirdita, Harald Voehringer, Stephan J. Haunsberger, Johannes Soeding. 2019. “HH-suite3 for fast remote homology detection and deep protein annotation.” *BMC Bioinformatics* 20: 473. <https://doi.org/10.1186/s12859-019-3019-7>

7. Wu, Ruonan, Eric M. Bottos, Vincent G. Danna, James C. Stegen, Janet K. Jansson, Michelle R. Davison. 2022. “RNA viruses linked to eukaryotic hosts in thawed permafrost.” *Msystems* 7: e00582-00522. <https://doi.org/10.1128/msystems.00582-22>

8. Luo, Xiao-Qing, Pandeng Wang, Jia-Ling Li, Manzoor Ahmad, Li Duan, Ling-Zi Yin, Qi-Qi Deng, et al. 2022. “Viral community-wide auxiliary metabolic genes differ by lifestyles, habitats, and hosts.” *Microbiome* 10: 190. <https://doi.org/10.1186/s40168-022-01384-y>

9. Yuan, Ling, Feng Ju. 2024. “Metatranscriptomic compendium of 55900 RNA viruses deciphers human health implication and ecological roles of RNA virome in global wastewater treatment plants.” *bioRxiv* <https://doi.org/10.1101/2024.03.12.584551>

10. Wu, Lilin, Yongqin Liu, Wenqing Shi, Tianyi Chang, Pengfei Liu, Keshao Liu, Yong He, et al. 2024. “Alpine lakes harbor diverse, endemic and structurally unique RNA viruses.” *bioRxiv* <https://doi.org/10.1101/2024.07.04.601995>

11. Liu, Yongqin, Zhihao Zhang, Nianzhi Jiao, Yongguan Zhu, Rui Zhang, Guillermo Dominguez-Huerta, Haina Wang, et al. 2024. “Diversity and ecological roles of RNA viral communities in the cryosphere of the Tibetan Plateau.” *bioRxiv* <https://doi.org/10.1101/2024.08.30.610425>

12. Sun, Jianhua, Kaiyang Zheng, Yan Liang, Mang Shi, Meiaoxue Han, Gang Liu, Chengxiang Gu, et al. 2022. “Diverse, abundant and stable coastal RNA viruses identified by spatiotemporal metatranscriptomics.” *Research Square* <https://doi.org/10.21203/rs.3.rs-2282285/v1>

13. Hyatt, Doug, Gwo-Liang Chen, Philip F. LoCascio, Miriam L. Land, Frank W. Larimer, Loren J. Hauser. 2010. “Prodigal: prokaryotic gene recognition and translation initiation site identification.” *BMC Bioinformatics* 11: 119. <https://doi.org/10.1186/1471-2105-11-119>

14. Babaian, Artem, Robert Edgar. 2022. “Ribovirus classification by a polymerase barcode sequence.” *Peerj* 10: e14055. <https://doi.org/10.7717/peerj.14055>

15. Zayed, Ahmed A., James M. Wainaina, Guillermo Dominguez-Huerta, Eric Pelletier, Jiarong Guo, Mohamed Mohssen, Funing Tian, et al. 2022. “Cryptic and abundant marine viruses at the evolutionary origins of Earth's RNA virome.” *Science* 376: 156-162. <https://doi.org/10.1126/science.abm5847>

16. Roux, Simon E., Antonio Pedro Camargo, Felipe H. Coutinho, Shareef M. Dabdoub, Bas E. Dutilh, Stephen Nayfach, Andrew Tritt. 2023. “iPHoP: an integrated machine learning framework to maximize host prediction for metagenome-derived viruses of archaea and bacteria.” *Plos Biology* 21: 4. <https://doi.org/10.1371/journal.pbio.3002083>

17. Shi, Mang, Xian-Dan Lin, Jun-Hua Tian, Liang-Jun Chen, Xiao Chen, Ci-Xiu Li, Xin-Cheng Qin, et al. 2016. “Redefining the invertebrate RNA virosphere.” *Nature* 540: 539-543. <https://doi.org/10.1038/nature20167>

18. Schulz, Frederik, Simon Roux, David Paez-Espino, Sean Jungbluth, David A. Walsh, Vincent J. Denef, Katherine D. McMahon, et al. 2020. “Giant virus diversity and host interactions through global metagenomics.” *Nature* 578: 432-436. <https://doi.org/10.1038/s41586-020-1957-x>

19. Jones, Philip, David Binns, Hsin-Yu Chang, Matthew Fraser, Weizhong Li, Craig McAnulla, Hamish McWilliam, et al. 2014. “InterProScan 5: genome-scale protein function classification.” *Bioinformatics* 30: 1236-1240. <https://doi.org/10.1093/bioinformatics/btu031>

20. Attwood, Teresa K., Alain Coletta, Gareth Muirhead, Athanasia Pavlopoulou, Peter B. Philippou, Ivan Popov, Carlos Romá-Mateo, et al. 2012. “The PRINTS database: a fine-grained protein sequence annotation and analysis resource-its status in 2012.” *Database-the Journal of Biological Databases and Curation* 2012: bas019. <https://doi.org/10.1093/database/bas019>

21. Kaell, Lukas, Anders Krogh, Erik L. L. Sonnhammer. 2007. “Advantages of combined transmembrane topology and signal peptide prediction -: the Phobius web server.” *Nucleic Acids Research* 35: W429-W432. <https://doi.org/10.1093/nar/gkm256>

22. Potenza, Emilio, Tomas Di Domenico, Ian Walsh, Silvio C. E. Tosatto. 2015. “MobiDB 2.0: an improved database of intrinsically disordered and mobile proteins.” *Nucleic Acids Research* 43: D315-D320. <https://doi.org/10.1093/nar/gku982>

23. Krogh, Anders, Björn Larsson, Gunnar von Heijne, Erik L.L Sonnhammer. 2001. “Predicting transmembrane protein topology with a hidden Markov model: application to complete genomes.” *Journal of Molecular Biology* 305: 567-580. <https://doi.org/10.1006/jmbi.2000.4315>

24. Mirdita, Milot, Lars von den Driesch, Clovis Galiez, Maria J. Martin, Johannes Soeding, Martin Steinegger. 2017. “Uniclust databases of clustered and deeply annotated protein sequences and alignments.” *Nucleic Acids Research* 45: D170-D176. <https://doi.org/10.1093/nar/gkw1081>

25. Finn, Robert D., Jody Clements, Sean R. Eddy. 2011. “HMMER web server: interactive sequence similarity searching.” *Nucleic Acids Research* 39: W29-W37. <https://doi.org/10.1093/nar/gkr367>

26. Lu, Shennan, Jiyao Wang, Farideh Chitsaz, Myra K. Derbyshire, Renata C. Geer, Noreen R. Gonzales, Marc Gwadz, et al. 2020. “CDD/SPARCLE: the conserved domain database in 2020.” *Nucleic Acids Research* 48: D265-D268. <https://doi.org/10.1093/nar/gkz991>

27. Mistry, Jaina, Sara Chuguransky, Lowri Williams, Matloob Qureshi, Gustavo A. Salazar, Erik L. L. Sonnhammer, Silvio C. E. Tosatto, et al. 2021. “Pfam: the protein families database in 2021.” *Nucleic Acids Research* 49: D412-D419. <https://doi.org/10.1093/nar/gkaa913>

28. Sillitoe, Ian, Nicola Bordin, Natalie Dawson, Vaishali P. Waman, Paul Ashford, Harry M. Scholes, Camilla S. M. Pang, et al. 2021. “CATH: increased structural coverage of functional space.” *Nucleic Acids Research* 49: D266-D273. <https://doi.org/10.1093/nar/gkaa1079>

29. Cheng, Hua, Yuxing Liao, R. Dustin Schaeffer, Nick V. Grishin. 2015. “Manual classification strategies in the ECOD database.” *Proteins-Structure Function and Bioinformatics* 83: 1238-1251. <https://doi.org/10.1002/prot.24818>

30. Edgar, Robert C. 2022. “Muscle5: High-accuracy alignment ensembles enable unbiased assessments of sequence homology and phylogeny.” *Nature Communications* 13: 6968. <https://doi.org/10.1038/s41467-022-34630-w>

31. Capella-Gutiérrez, Salvador, Jose M. Silla-Martínez, Toni Gabaldón. 2009. “trimAl: a tool for automated alignment trimming in large-scale phylogenetic analyses.” *Bioinformatics* 25: 1972-1973. <https://doi.org/10.1093/bioinformatics/btp348>

32. Tamura, Koichiro, Glen Stecher, Daniel Peterson, Alan Filipski, Sudhir Kumar. 2013. “MEGA6: molecular evolutionary genetics analysis version 6.0.” *Molecular Biology and Evolution* 30: 2725-2729. <https://doi.org/10.1093/molbev/mst197>

33. Hall, Barry G. 2013. “Building phylogenetic trees from molecular data with MEGA.” *Molecular Biology and Evolution* 30: 1229-1235. <https://doi.org/10.1093/molbev/mst012>

34. Letunic, Ivica, Peer Bork. 2021. “Interactive Tree Of Life (iTOL) v5: an online tool for phylogenetic tree display and annotation.” *Nucleic Acids Research* 49: W293-W296. <https://doi.org/10.1093/nar/gkab301>


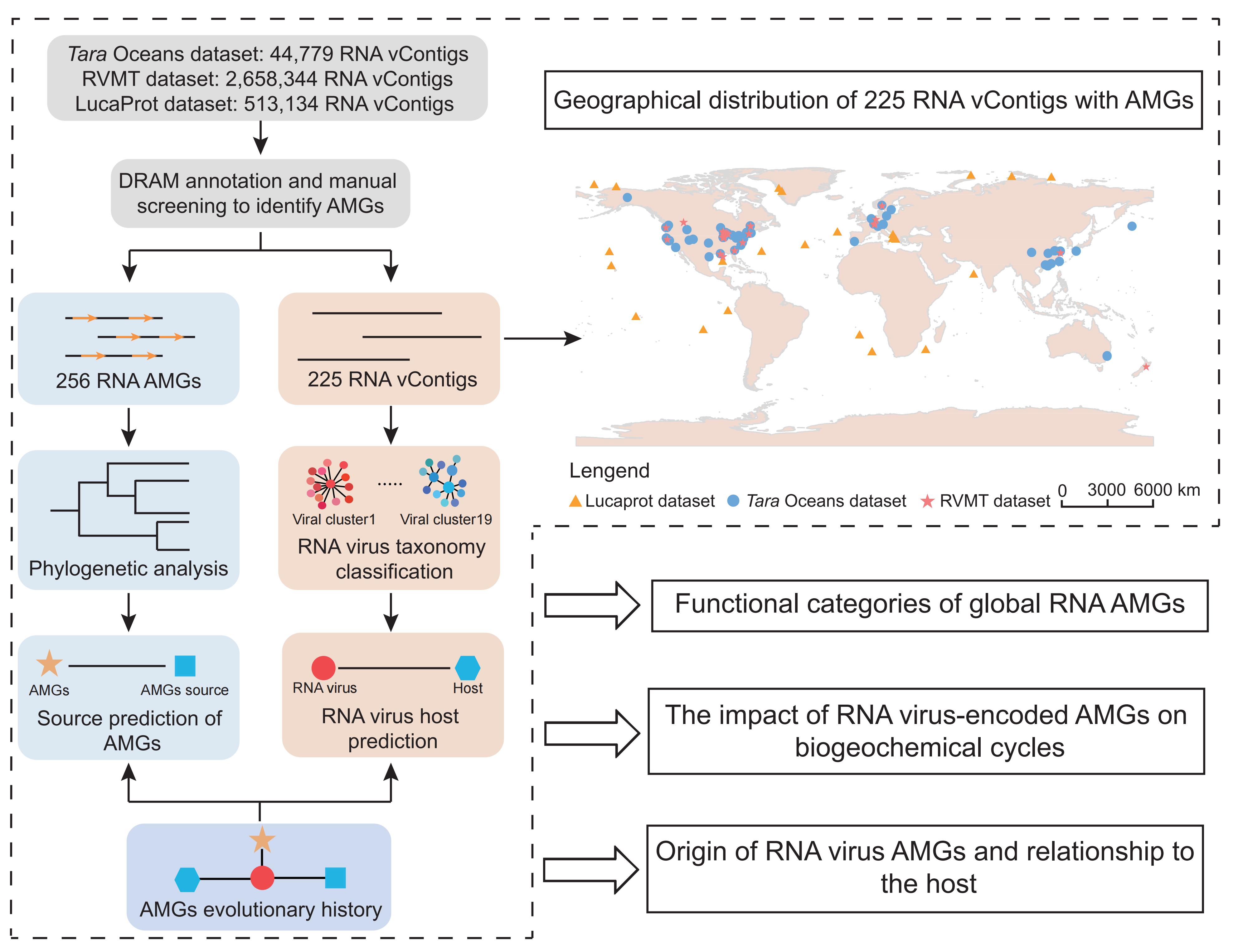


**Figure S1 Overview of the global RNA viral AMGs analysis pipeline.** The diagram outlines the workflow from global RNA virus genome data collection and processing to the analysis of RNA viral AMGs. 3,216,257 RNA vContigs from 16,408 metatranscriptomes were collected from the *Tara* Oceans, RVMT, and LucaProt datasets to study RNA viral AMGs. Host prediction for RNA vContigs and phylogenetic analysis of AMGs suggested the evolutionary history of selected AMGs and potential virus-host interactions.


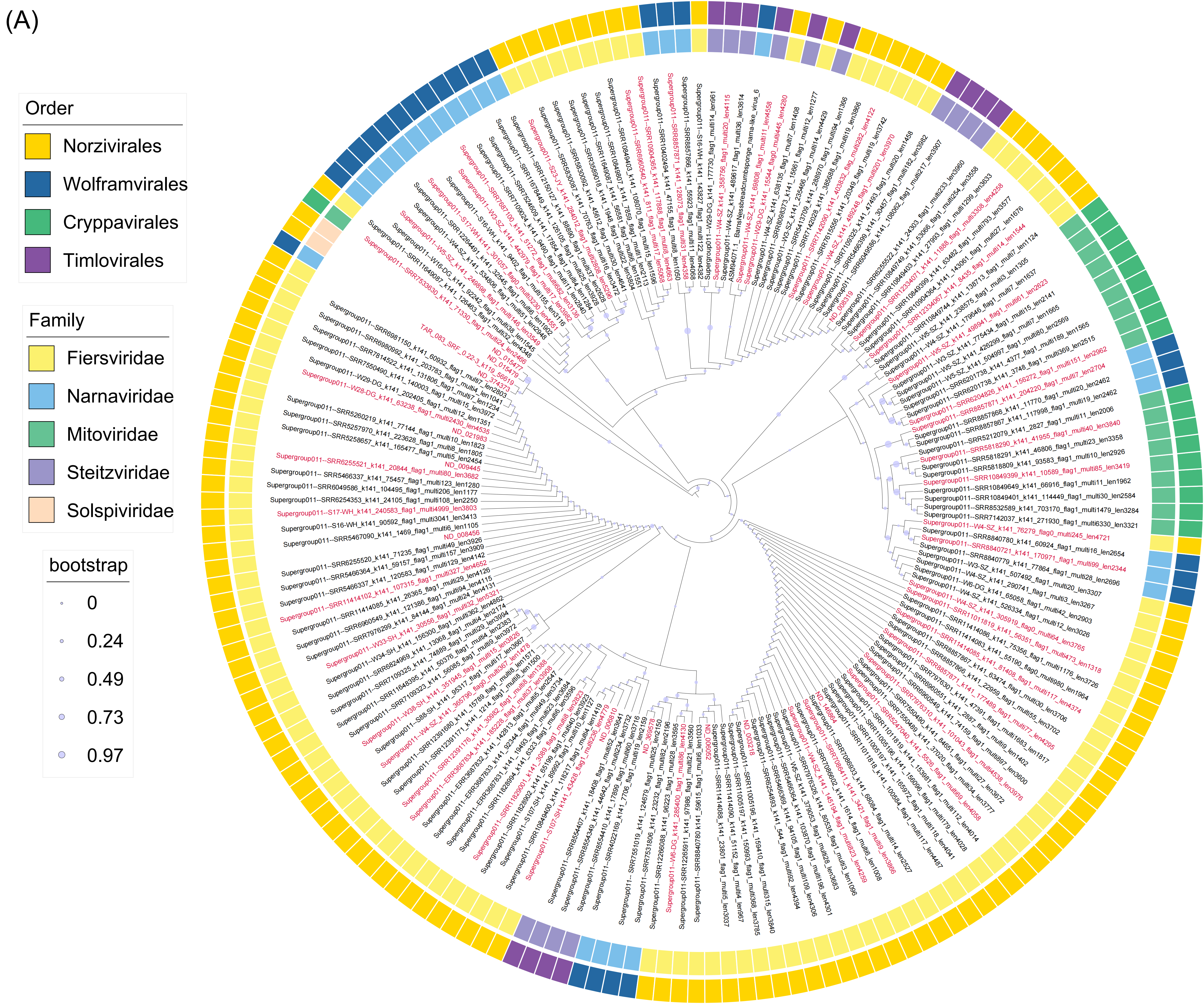


**Figure S2 Phylogenetic tree of RdRps for four phyla.** (A) Phylogenetic tree of RdRps for the Lenarviricota phylum. RNA viral RdRPs with AMGs are highlighted in red, while reference sequences are highlighted in black. The outer ring shows the order and family of RdRps, respectively.


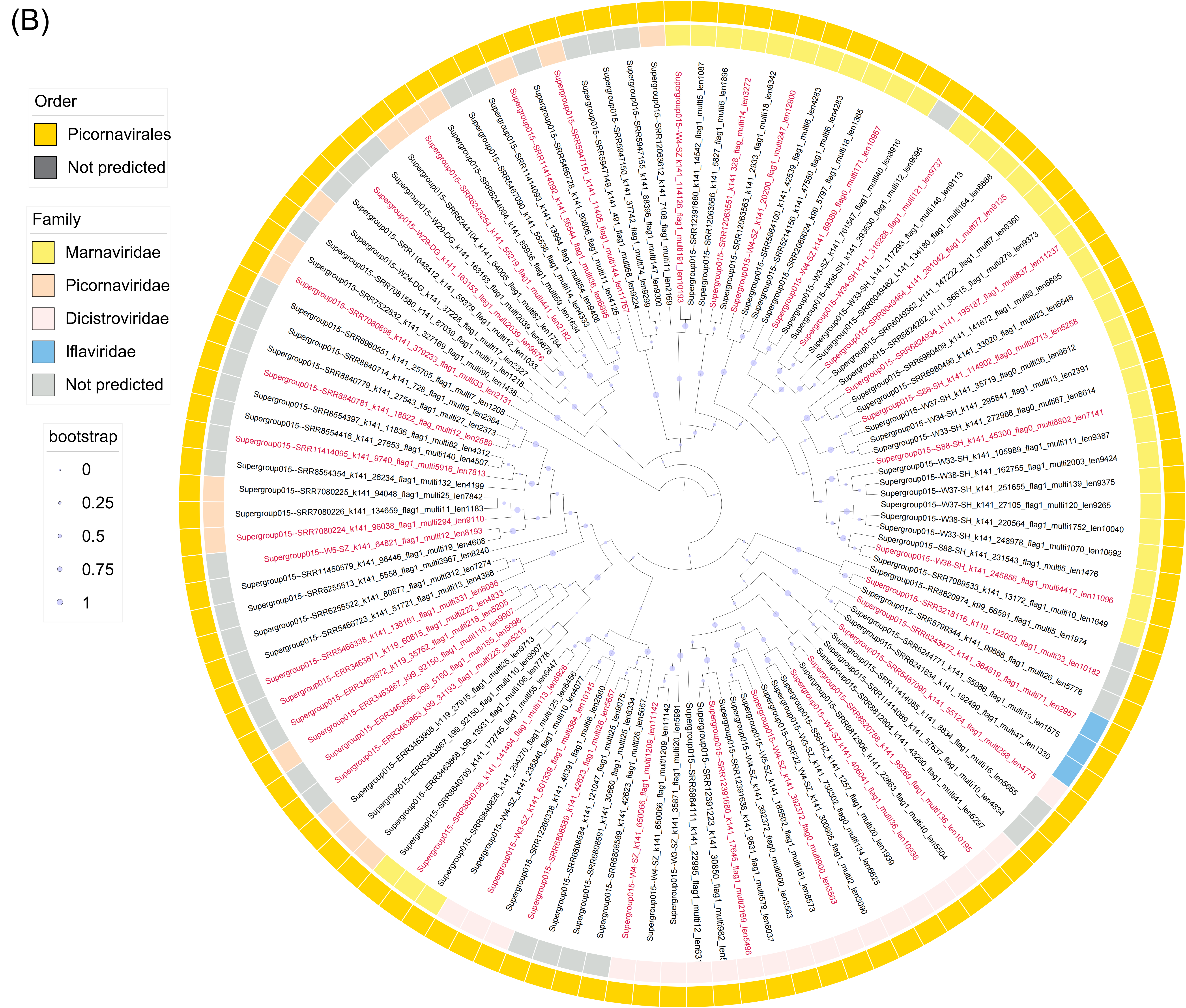


**Figure S2 Phylogenetic tree of RdRps for four phyla.** (B) Phylogenetic tree of RdRps for the picorna supergroup phylum. RNA viral RdRPs with AMGs are highlighted in red, while reference sequences are highlighted in black. The outer ring shows the order and family of RdRps, respectively.


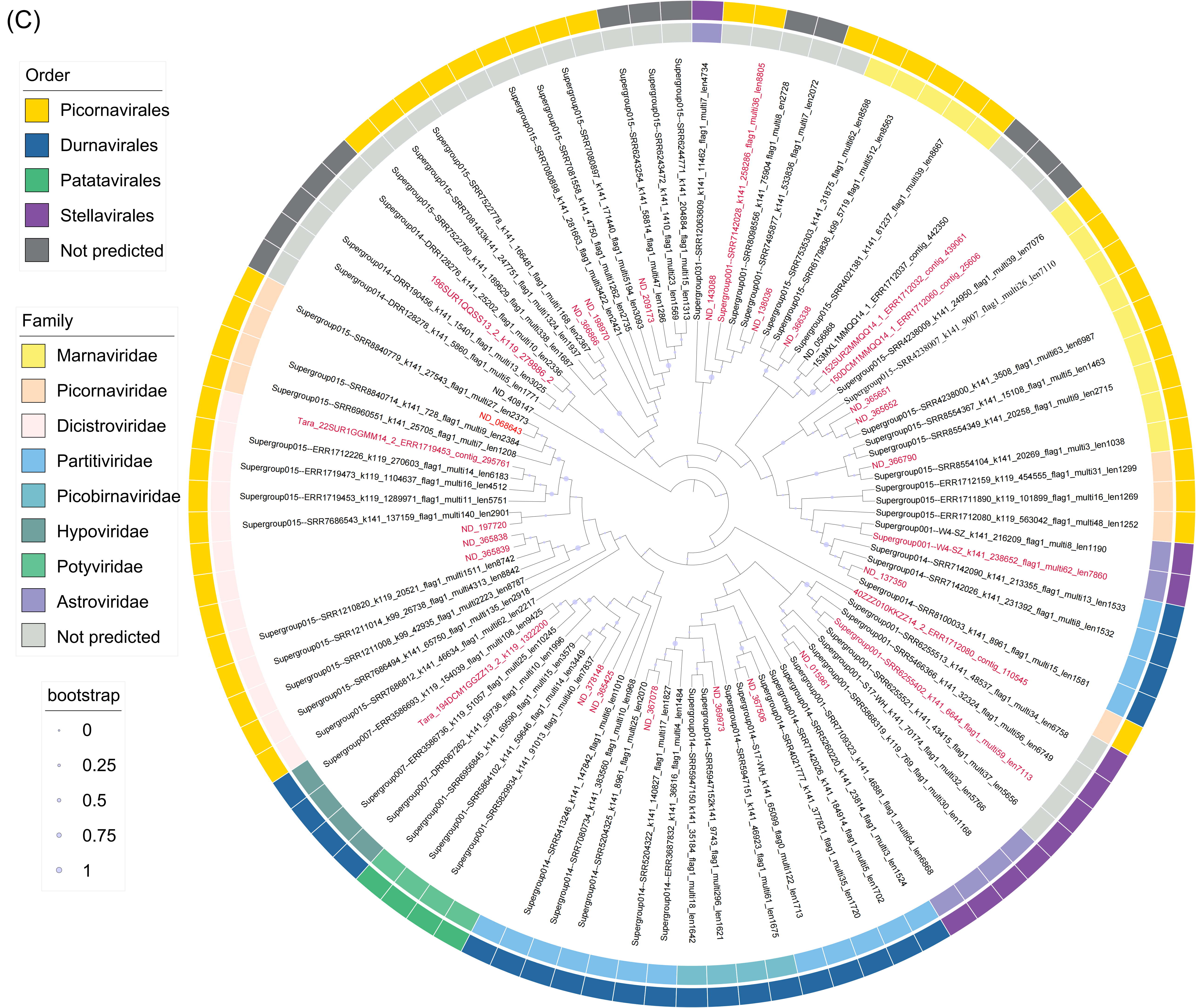


**Figure S2 Phylogenetic tree of RdRps for four phyla.** (C) Phylogenetic tree of RdRps for the Pisuviricota phylum. RNA viral RdRPs with AMGs are highlighted in red, while reference sequences are highlighted in black. The outer ring shows the order and family of RdRps, respectively.


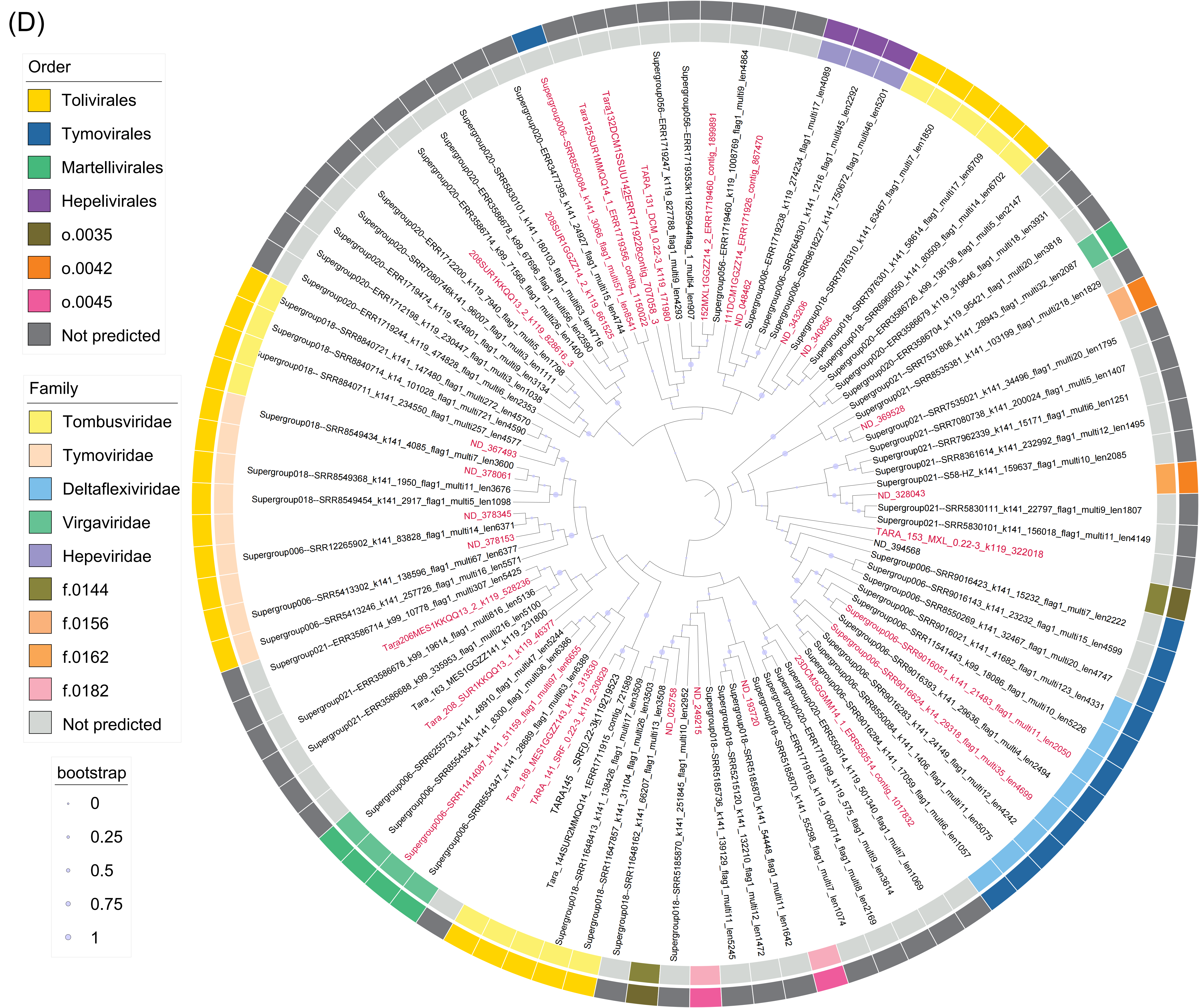


**Figure S2 Phylogenetic tree of RdRps for four phyla.** (D) Phylogenetic tree of RdRps for the Kitrinoviricota phylum. RNA viral RdRPs with AMGs are highlighted in red, while reference sequences are highlighted in black. The outer ring shows the order and family of RdRps, respectively.


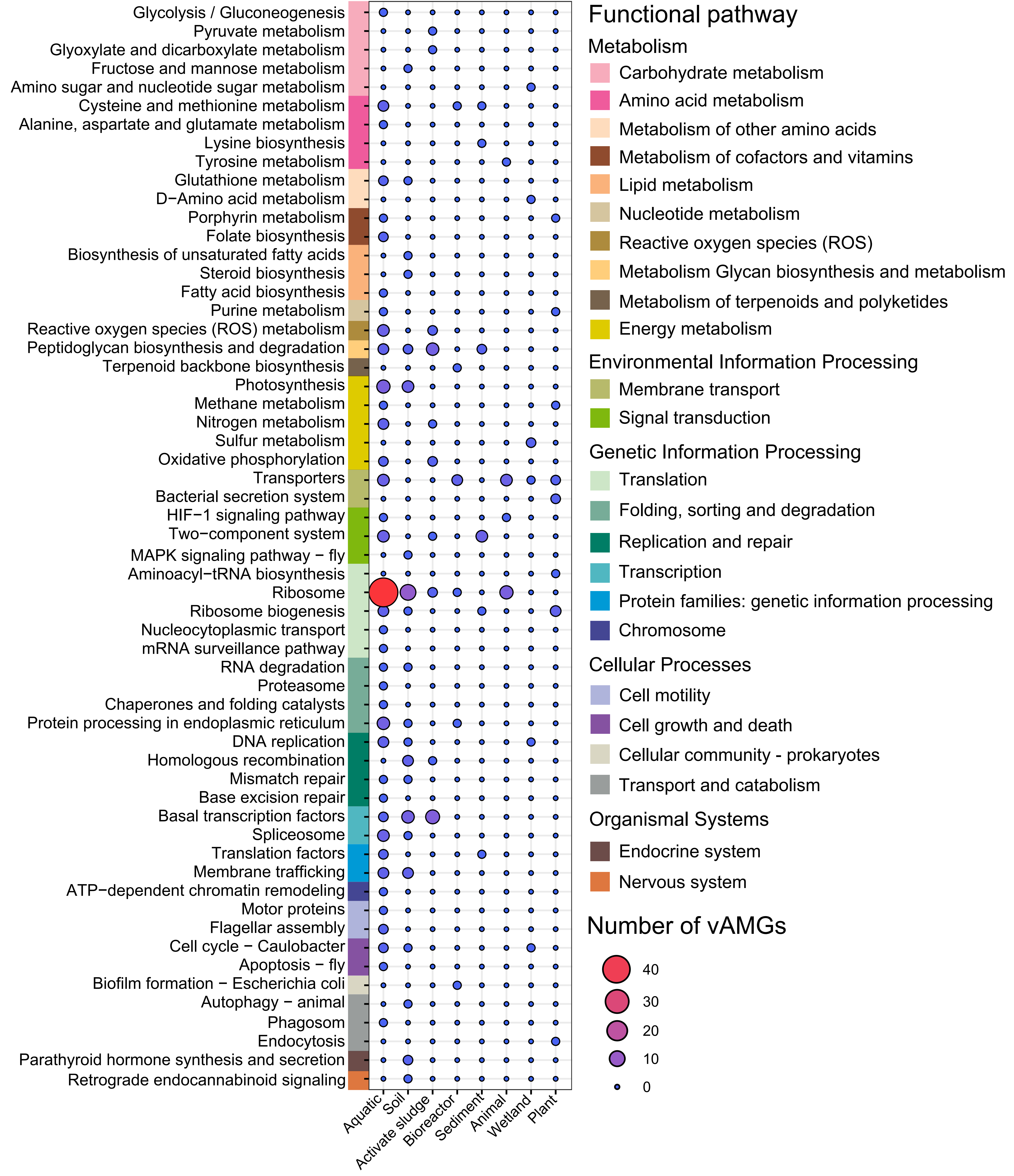


**Figure S3 RNA viral AMGs are distributed in eight habitats and the 58 biological pathways.**


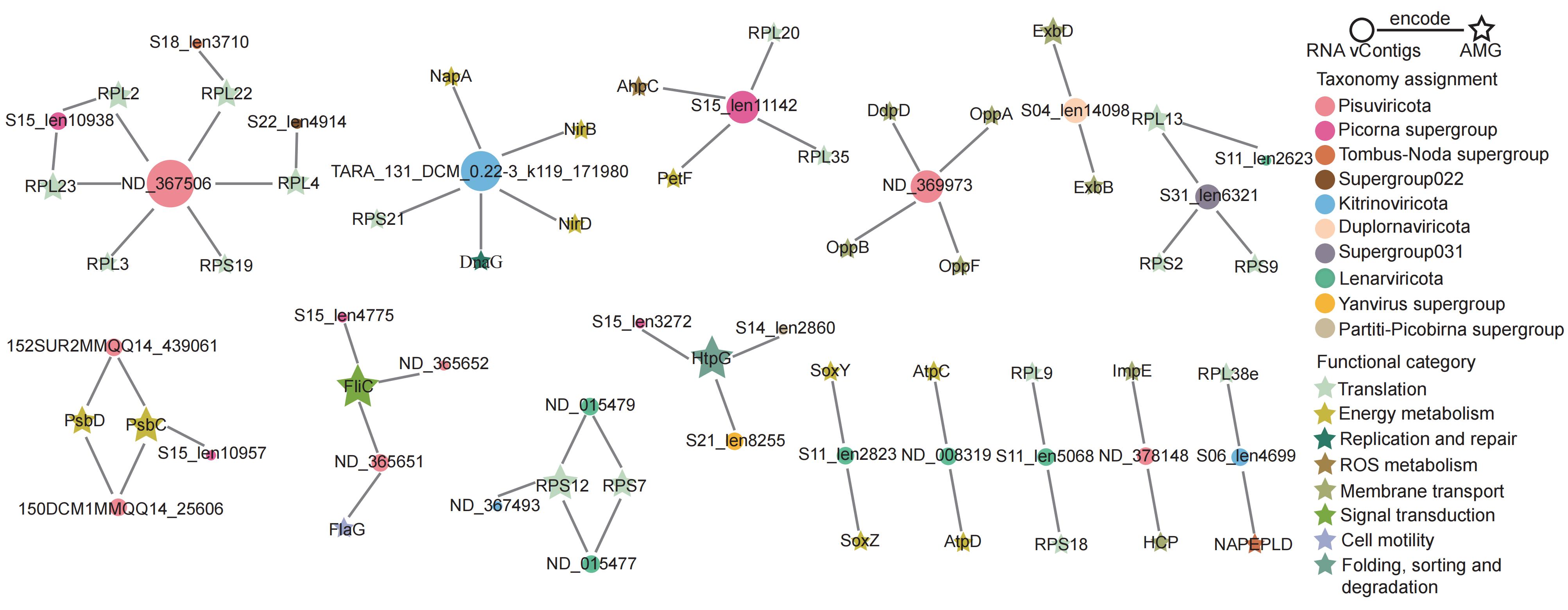


**Figure S4 Network diagram of 18 RNA vContigs encoding multiple AMGs.** Each node represents an vContigs or an AMG, and the color of the node represents the taxonomy of vContigs or the pathway that the AMG is involved in. Each line indicates the vContig encoding the connected AMG.


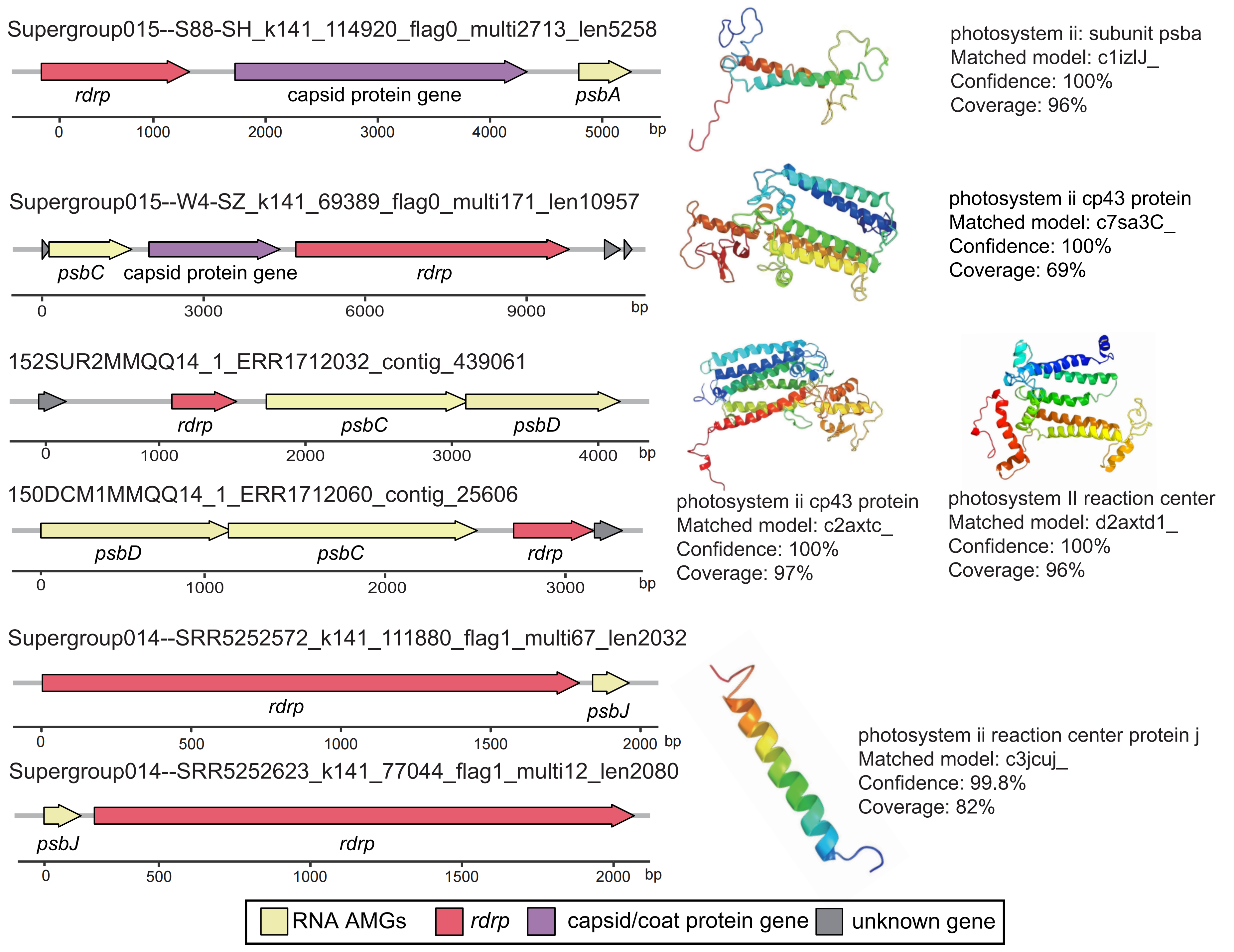


**Figure S5 The AMGs encoded by RNA vContigs revealed the involvement of RNA viruses in photosynthesis system.** The genome architecture of RNA vContigs encoding *psbA*, *psbC*, *psbD*, and *psbJ* are on the left, and the predictive structure model of the proteins corresponding to these genes is shown on the right. 152SUR2MMQQ14_1_ERR1712032_contig_439061 and 150DCM1MMQQ14_1_ERR1712060_contig_25606 share the same PsbC and PsbD protein structure models. Supergroup014--SRR5252572_k141_111880_flag1_multi67_len2032 and Supergroup014--SRR5252623_k141_77044_flag1_multi12_len2080 share the same PsbJ protein structure models.


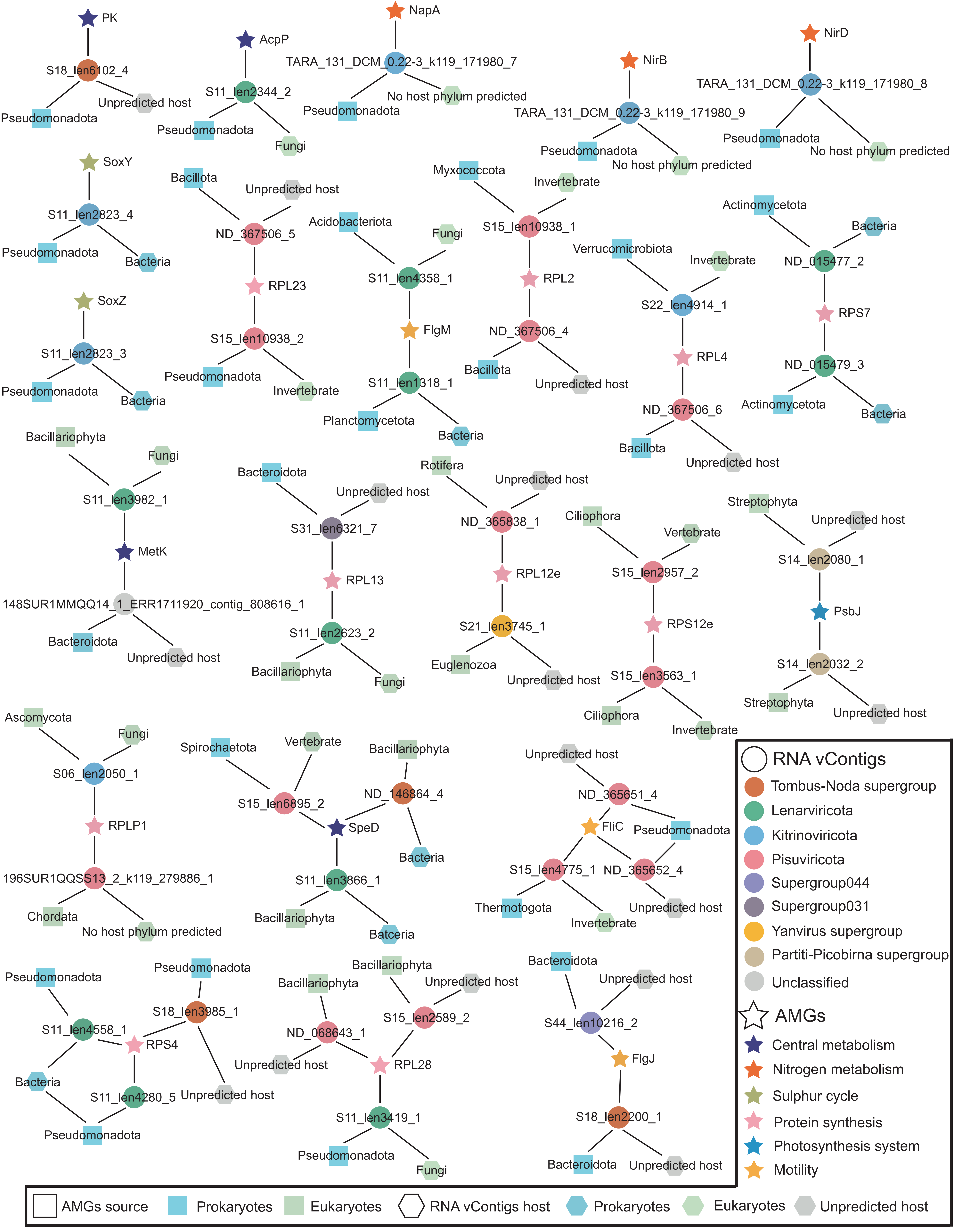


**Figure S6 Network diagram of RNA vContigs with host information and their AMGs sources, including AMGs related to biogeochemical cycling, protein synthesis, and motility.** Circles represent RNA vContigs encoding AMGs, pentagons represent AMGs, squares represent probable sources of AMGs as predicted by the phylogenetic tree, and hexagons represent hosts that RNA vContigs may infect.


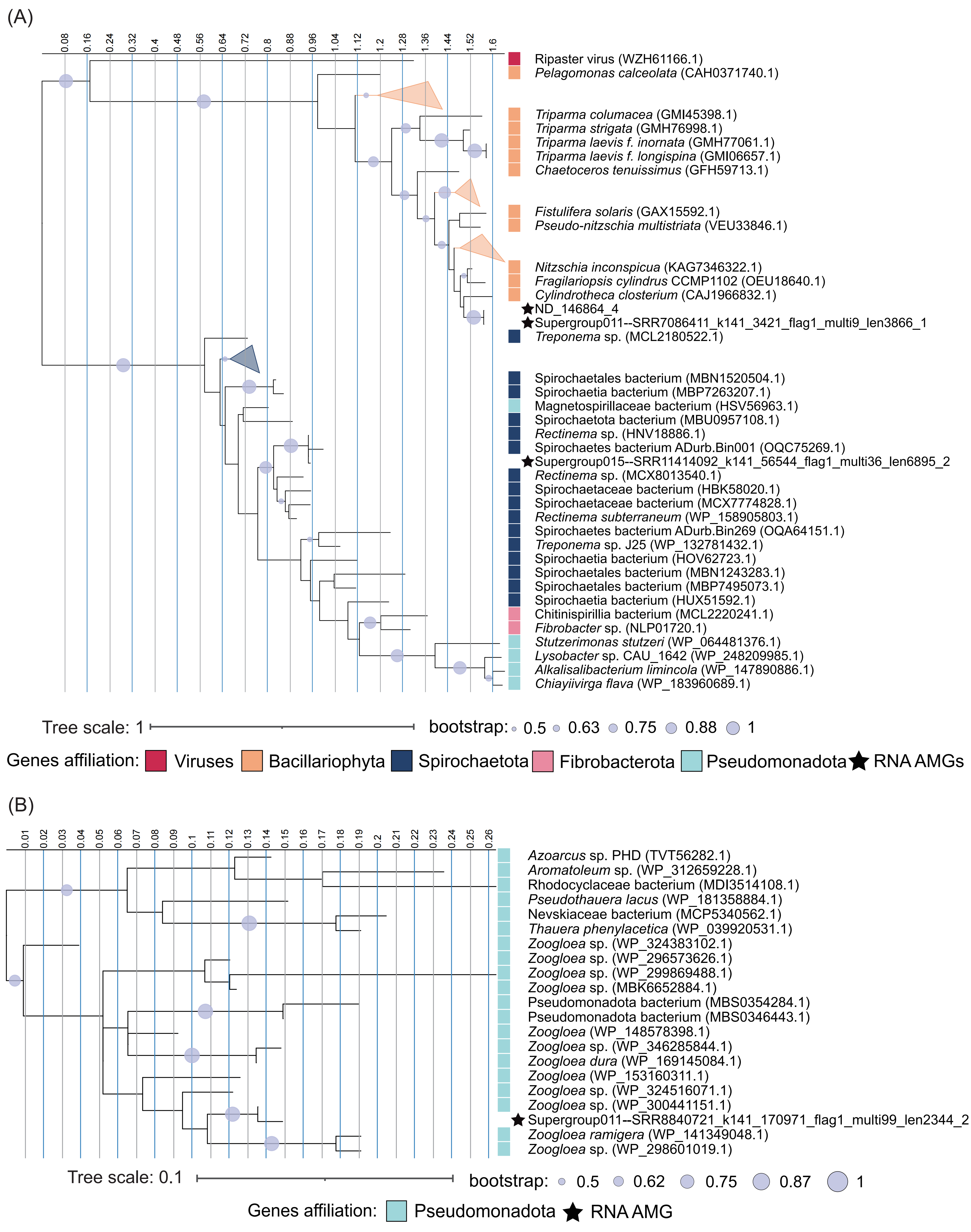


**Figure S7 Phylogenetic tree of RNA AMGs that may have originated in prokaryotes.** (A) Phylogenetic tree of RNA viral *speD* and reference *speD* sequences found in NCBI nr database. (B) Phylogenetic tree of RNA viral *acpP* and reference *acpP* sequences found in NCBI nr database.


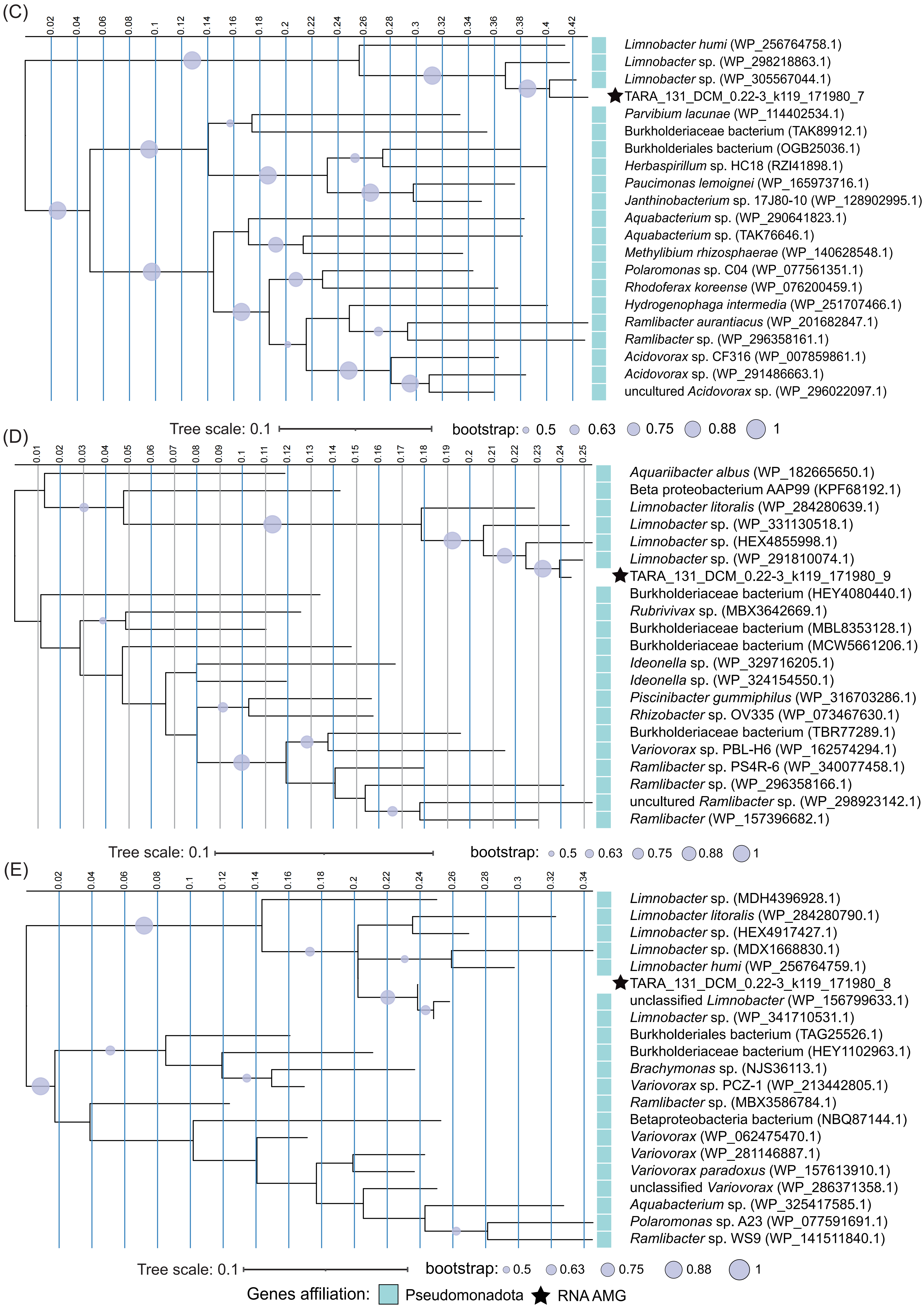


**Figure S7 Phylogenetic tree of RNA AMGs that may have originated in prokaryotes.** (C) Phylogenetic tree of RNA viral *napA* and reference *napA* sequences found in NCBI nr database. (D) Phylogenetic tree of RNA viral *nirB* and reference *nirB* sequences found in NCBI nr database. (E) Phylogenetic tree of RNA viral *nirD* and reference *nirD* sequences found in NCBI nr database.


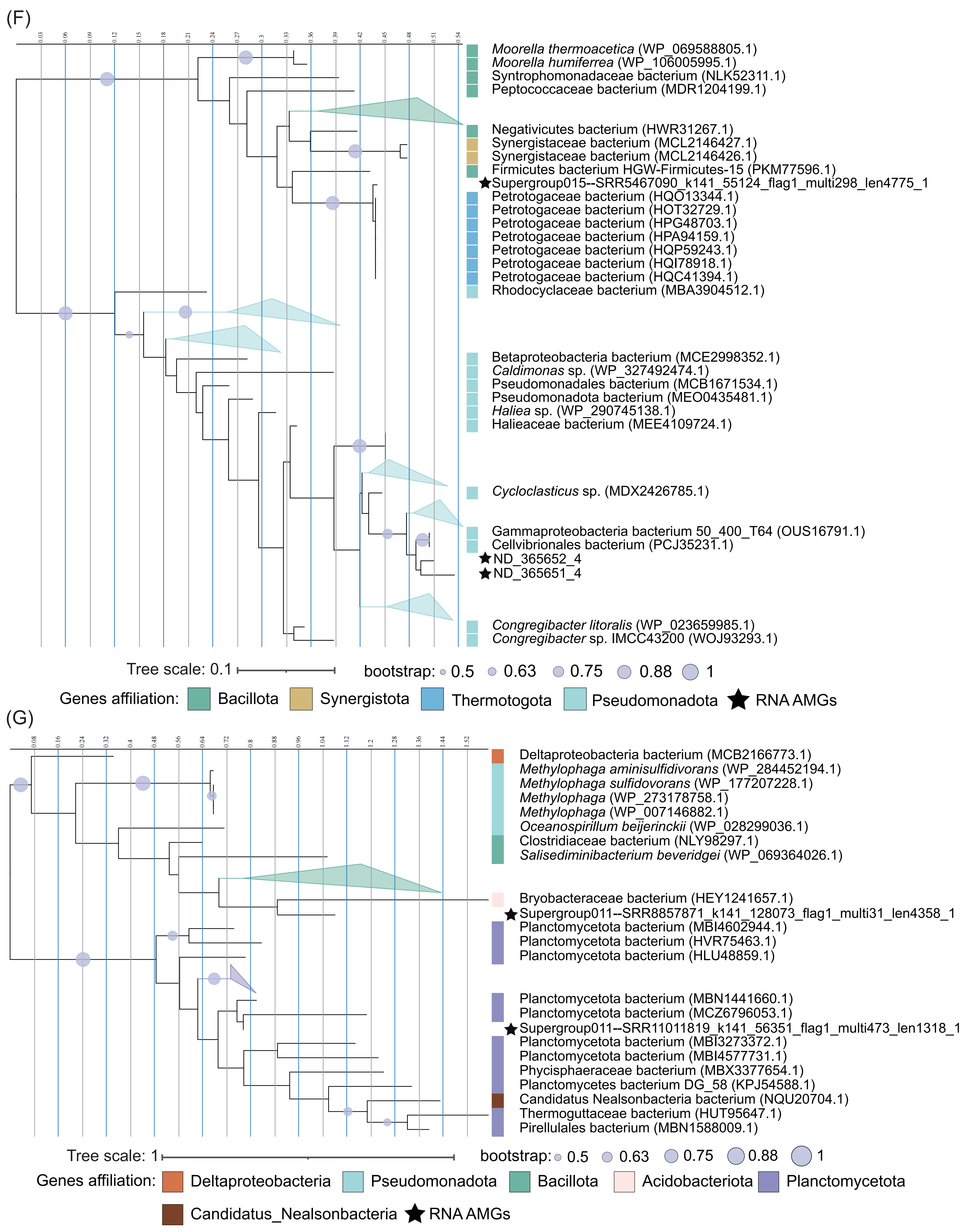


**Figure S7 Phylogenetic tree of RNA AMGs that may have originated in prokaryotes.** (F) Phylogenetic tree of RNA viral *fliC* and reference *fliC* sequences found in NCBI nr database. (G) Phylogenetic tree of RNA viral *flgM* and reference *flgM* sequences found in NCBI nr database.


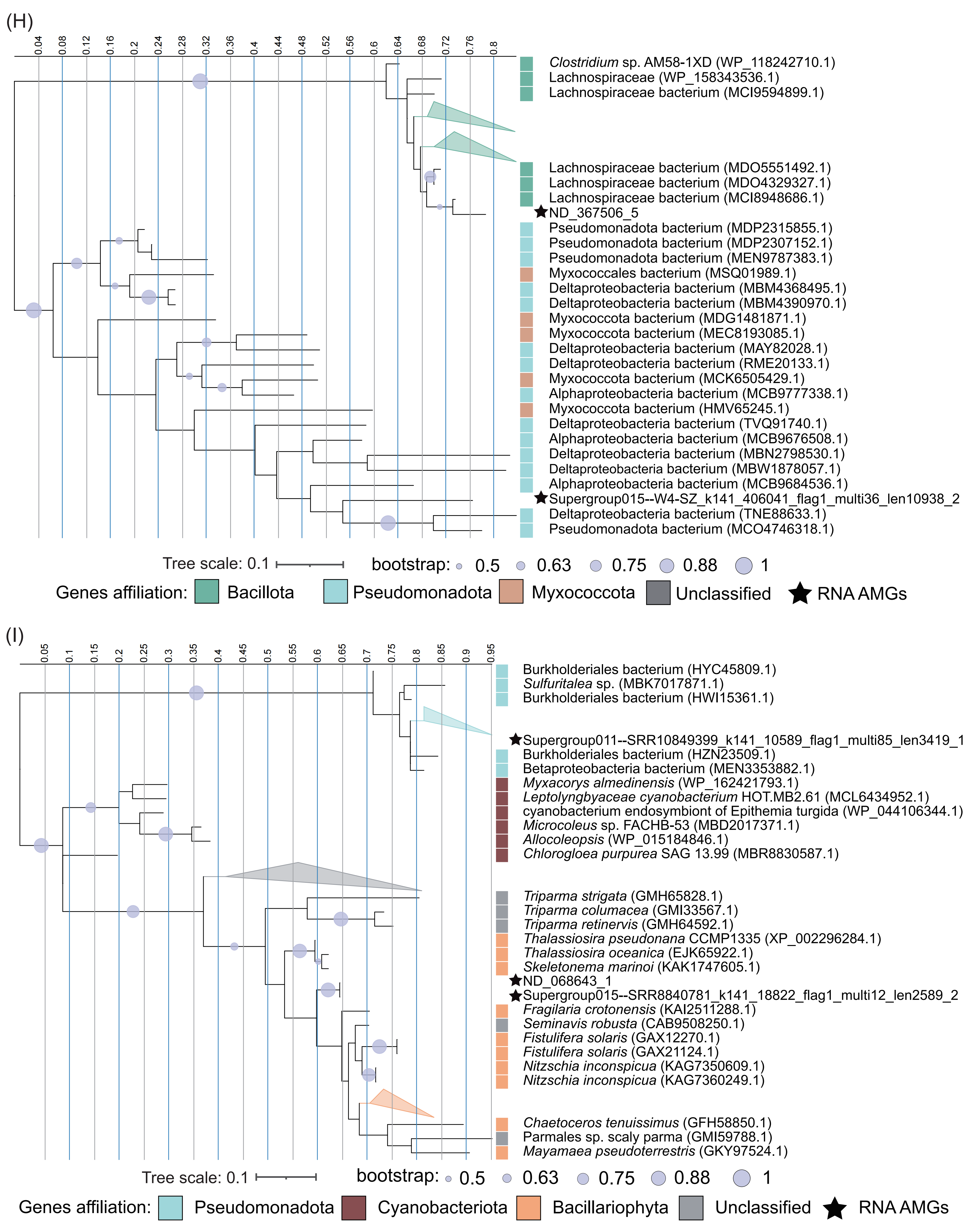


**Figure S7 Phylogenetic tree of RNA AMGs that may have originated in prokaryotes.** (H) Phylogenetic tree of RNA viral *rpl23* and reference *rpl23* sequences found in NCBI nr database. (I) Phylogenetic tree of RNA viral *rpl28* and reference *rpl28* sequences found in NCBI nr database.


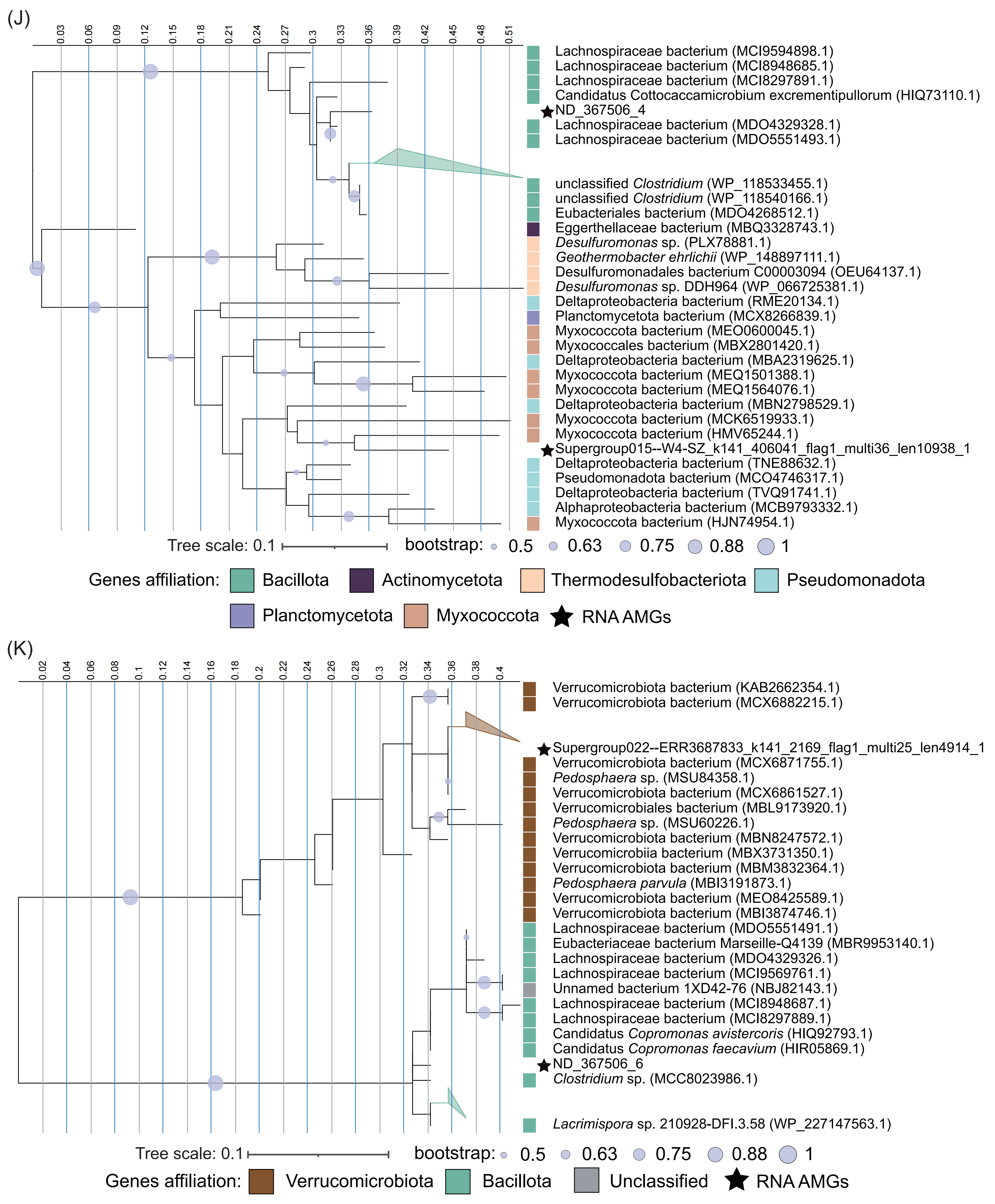


**Figure S7 Phylogenetic tree of RNA AMGs that may have originated in prokaryotes.** (J) Phylogenetic tree of RNA viral *prl2* and reference *rpl2* sequences found in NCBI nr database. (K) Phylogenetic tree of RNA viral *rpl4* and reference *rpl4* sequences found in NCBI nr database.


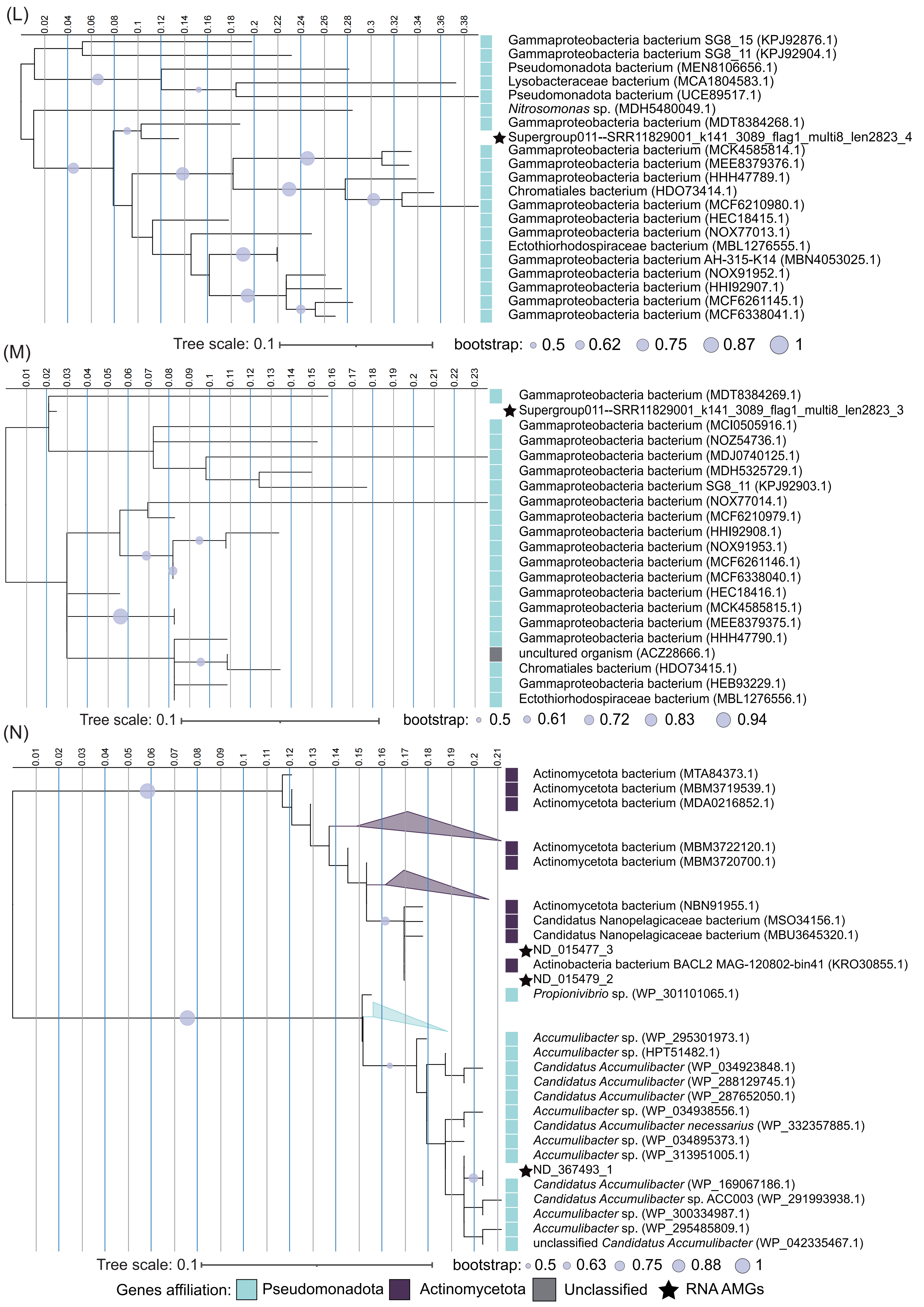


**Figure S7 Phylogenetic tree of RNA AMGs that may have originated in prokaryotes.** (L) Phylogenetic tree of RNA viral *soxY* and reference *soxY* sequences found in NCBI nr database. (M) Phylogenetic tree of RNA viral *soxZ* and reference *soxZ* sequences found in NCBI nr database. (N) Phylogenetic tree of RNA viral *rps12* and reference *rps12* sequences found in NCBI nr database.


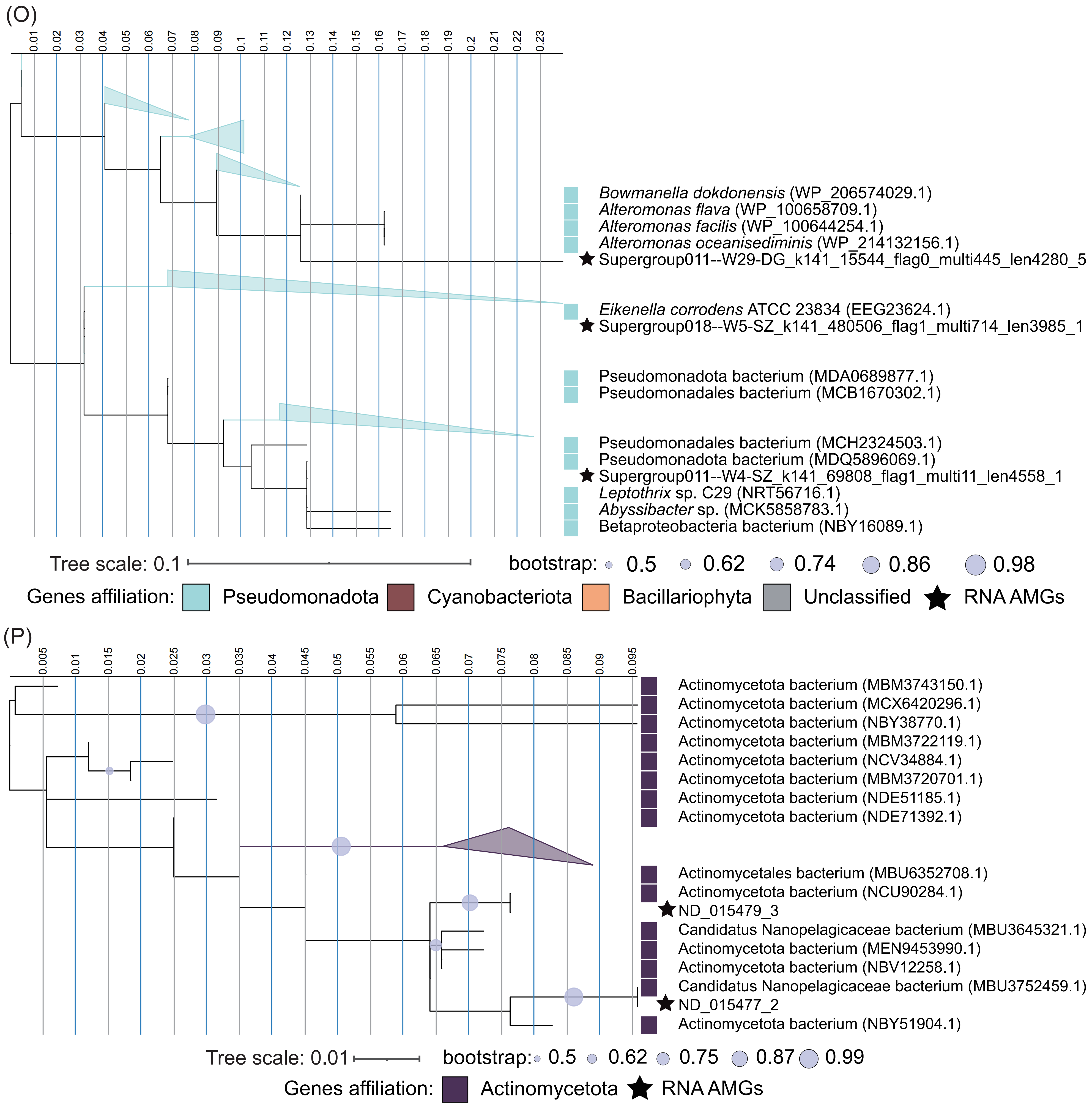


**Figure S7 Phylogenetic tree of RNA AMGs that may have originated in prokaryotes.** (O) Phylogenetic tree of RNA viral *rps4* and reference *rps4* sequences found in NCBI nr database. (P) Phylogenetic tree of RNA viral *rps7* and reference *rps7* sequences found in NCBI nr database.


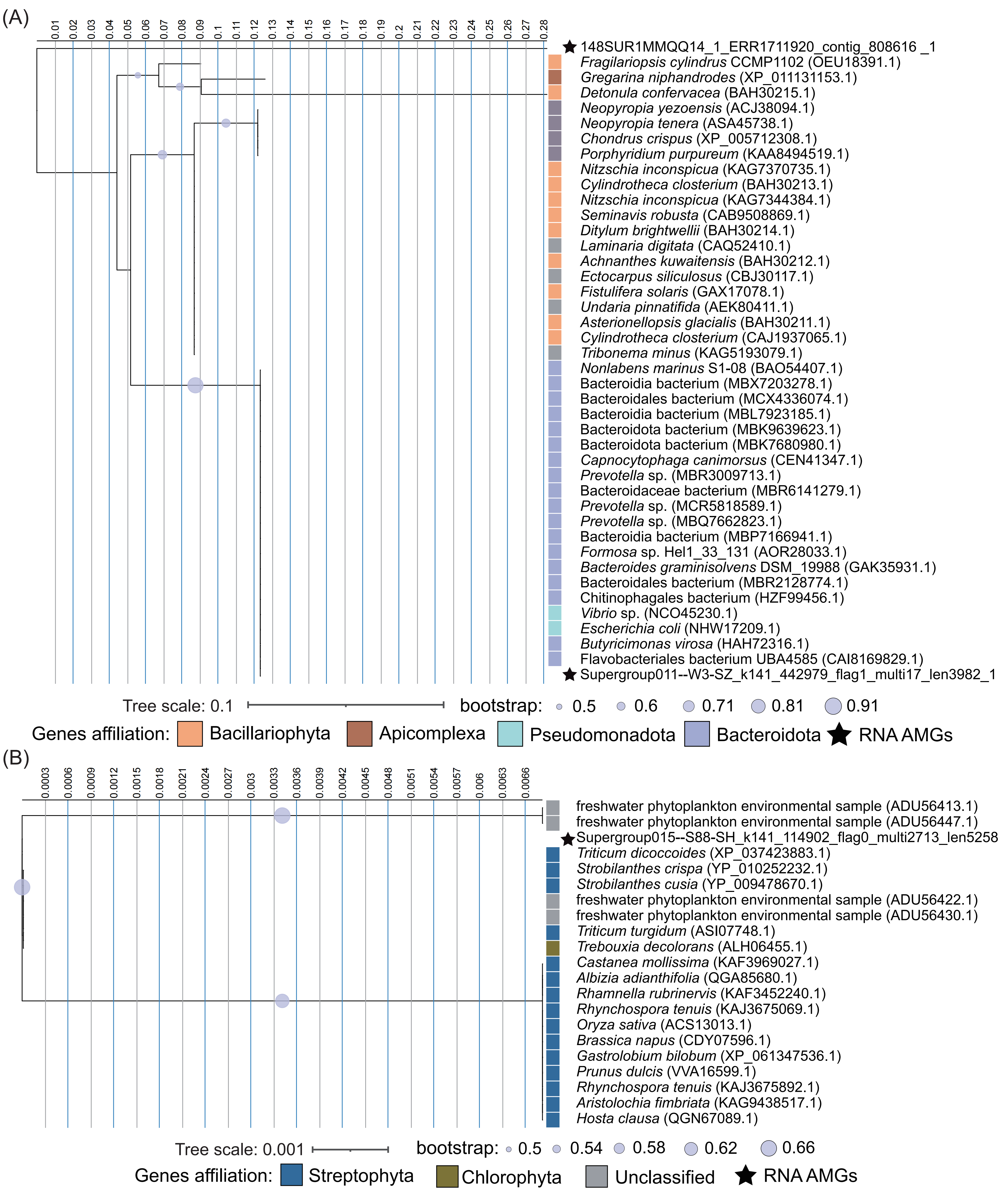


**Figure S8 Phylogenetic tree of RNA AMGs that may have originated in eukaryotes.** (A) Phylogenetic tree of RNA viral *metK* and reference *metK* sequences found in NCBI nr database. (B) Phylogenetic tree of RNA viral *psbA* and reference *psbA* sequences found in NCBI nr database.


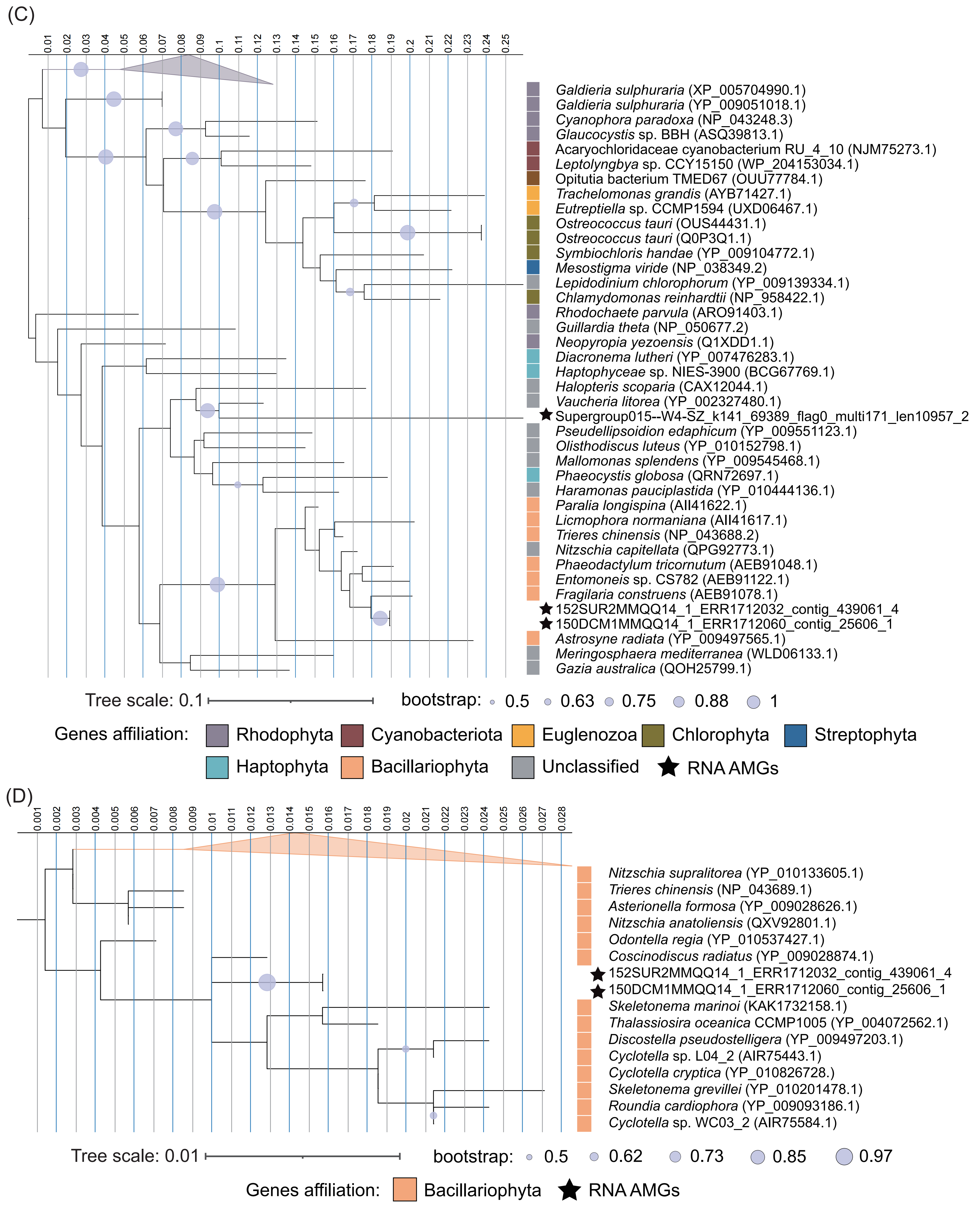


**Figure S8 Phylogenetic tree of RNA AMGs that may have originated in eukaryotes.** (C) Phylogenetic tree of RNA viral *psbC* and reference *psbC* sequences found in NCBI nr database. (D) Phylogenetic tree of RNA viral *psbD* and reference *psbD* sequences found in NCBI nr database.


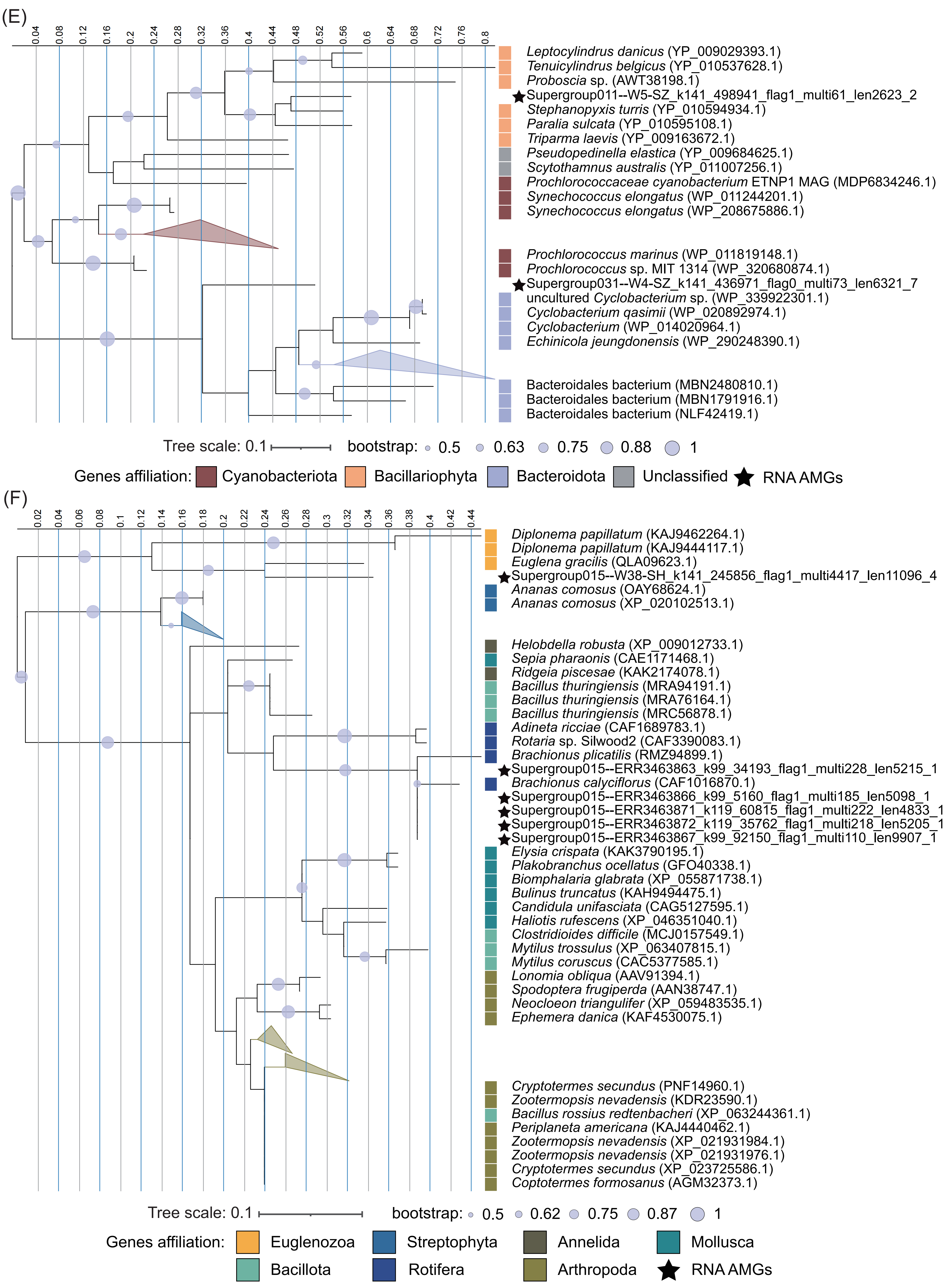


**Figure S8 Phylogenetic tree of RNA AMGs that may have originated in eukaryotes.** (E) Phylogenetic tree of RNA viral *rpl13* and reference *rpl13* sequences found in NCBI nr database. (F) Phylogenetic tree of RNA viral *rpl19e* and reference *rpl19e* sequences found in NCBI nr database.


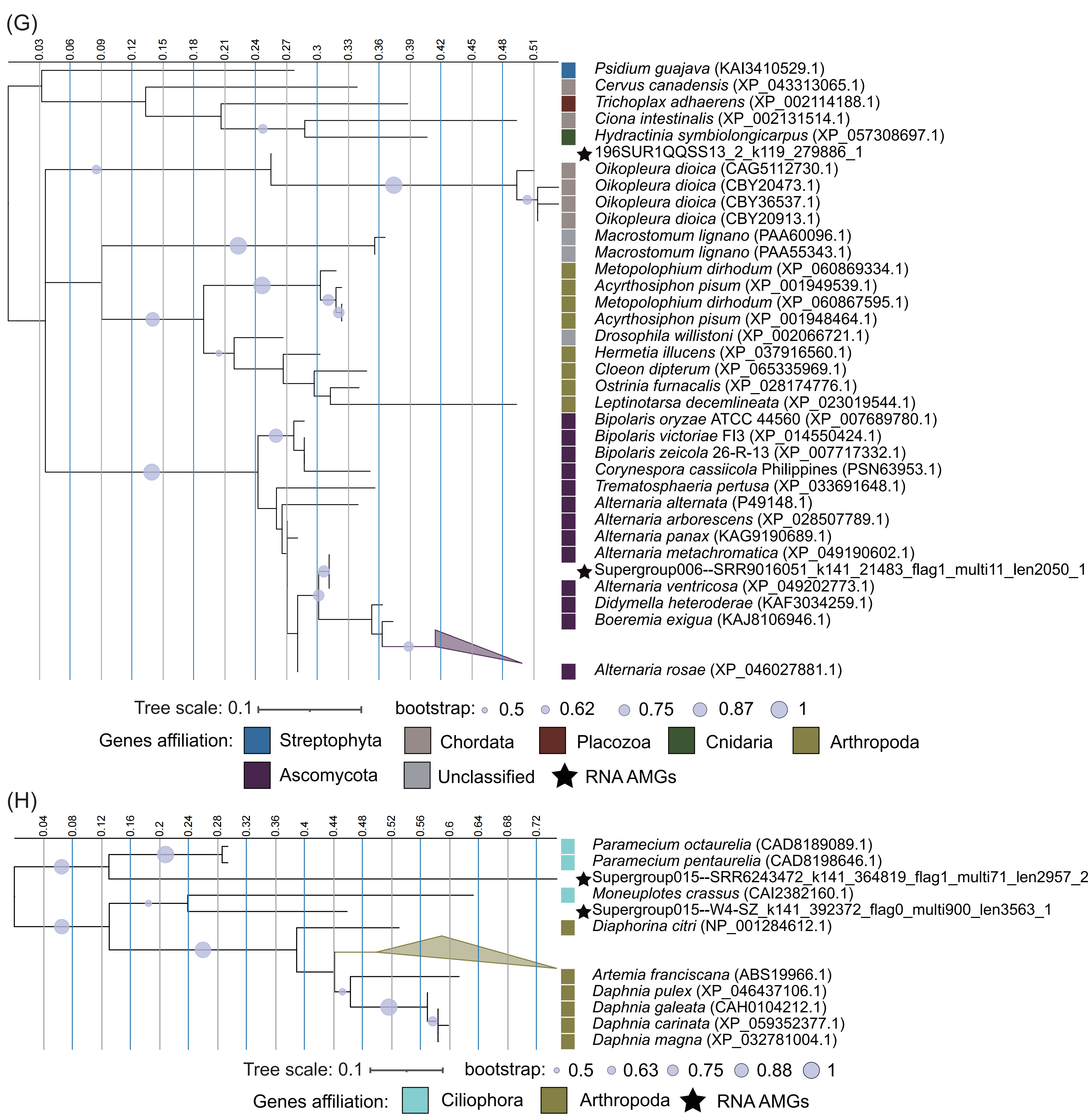


**Figure S8 Phylogenetic tree of RNA AMGs that may have originated in eukaryotes.** (G) Phylogenetic tree of RNA viral *rplP1* and reference *rplP1* sequences found in NCBI nr database. (H) Phylogenetic tree of RNA viral *rps12e* and reference *rps12e* sequences found in NCBI nr database.


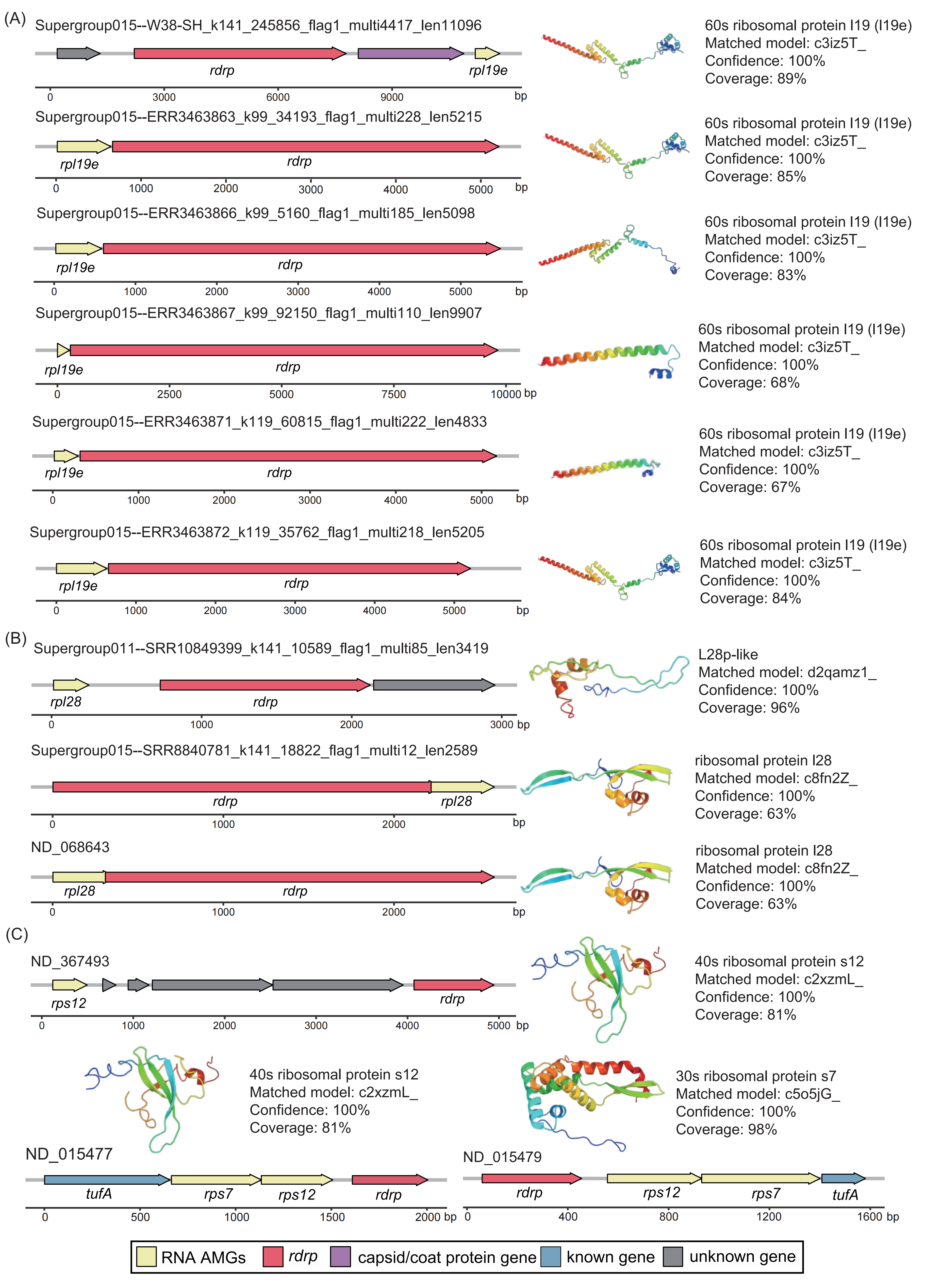


**Figure S9 Genome architecture of RNA vContig encoding a ribosomal protein gene and reference protein model.** (A) The genome architecture of RNA vContig encoding *rpl19e* gene and reference protein model for RPL19e. (B) Genome architecture of RNA vContig encoding *rpl28* gene and reference protein model for RPL28. (C) Genome architecture of RNA vContig encoding *rps12* gene and reference protein model for RPS12.


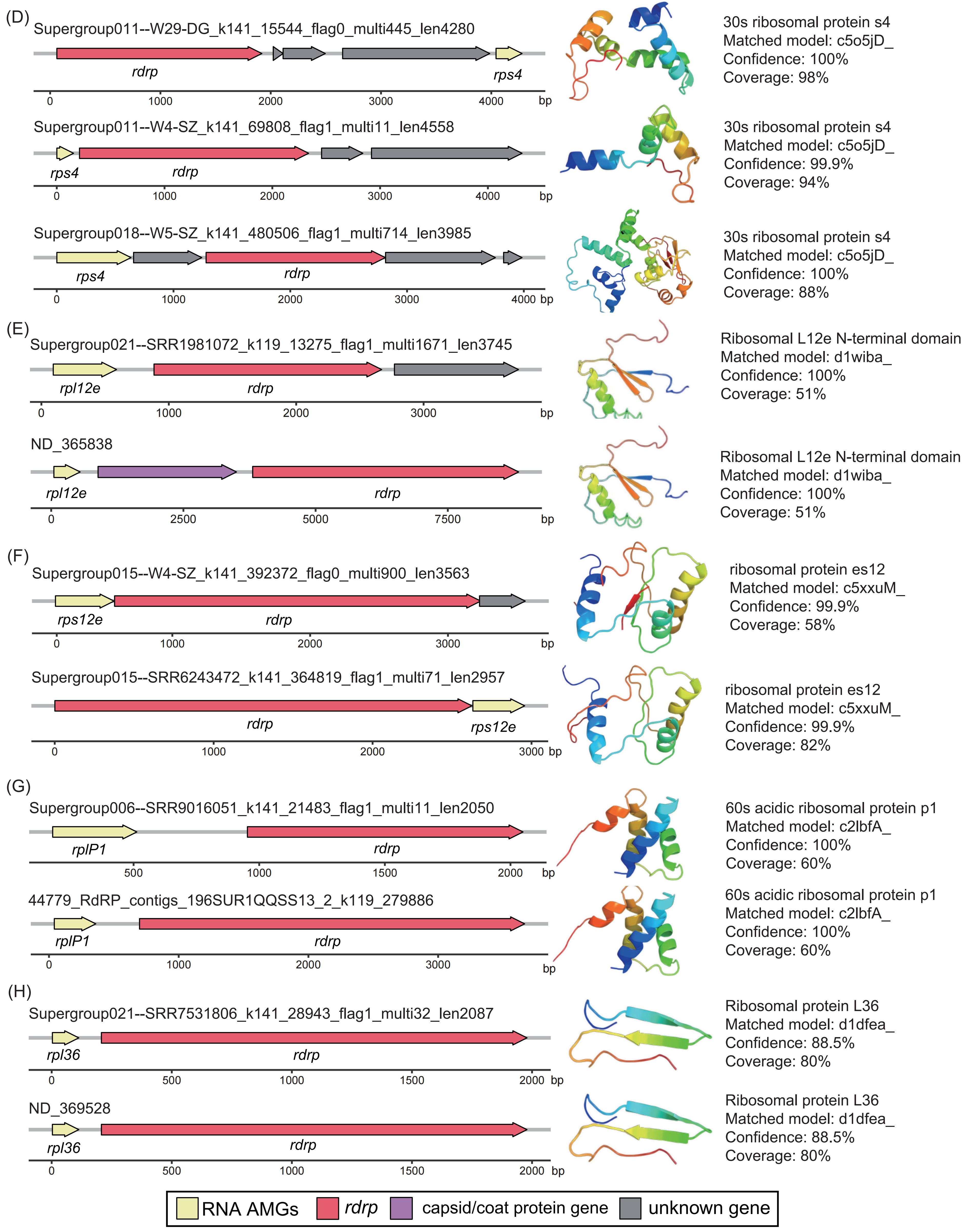


**Figure S9 Genome architecture of RNA vContig encoding a ribosomal protein gene and reference protein model.** (D) Genome architecture of RNA vContig encoding *rps4* gene and reference protein model for RPS4. (E) Genome architecture of RNA vContig encoding *rpl28* gene and reference protein model for PRL28. (F) Genome architecture of RNA vContig encoding *rps12e* gene and reference protein model for RPS12e. (G) Genome architecture of RNA vContig encoding *rplP1* gene and reference protein model for LPLP1. (H) Genome architecture of RNA vContig encoding *rpl36* gene and reference protein model for RPL36.


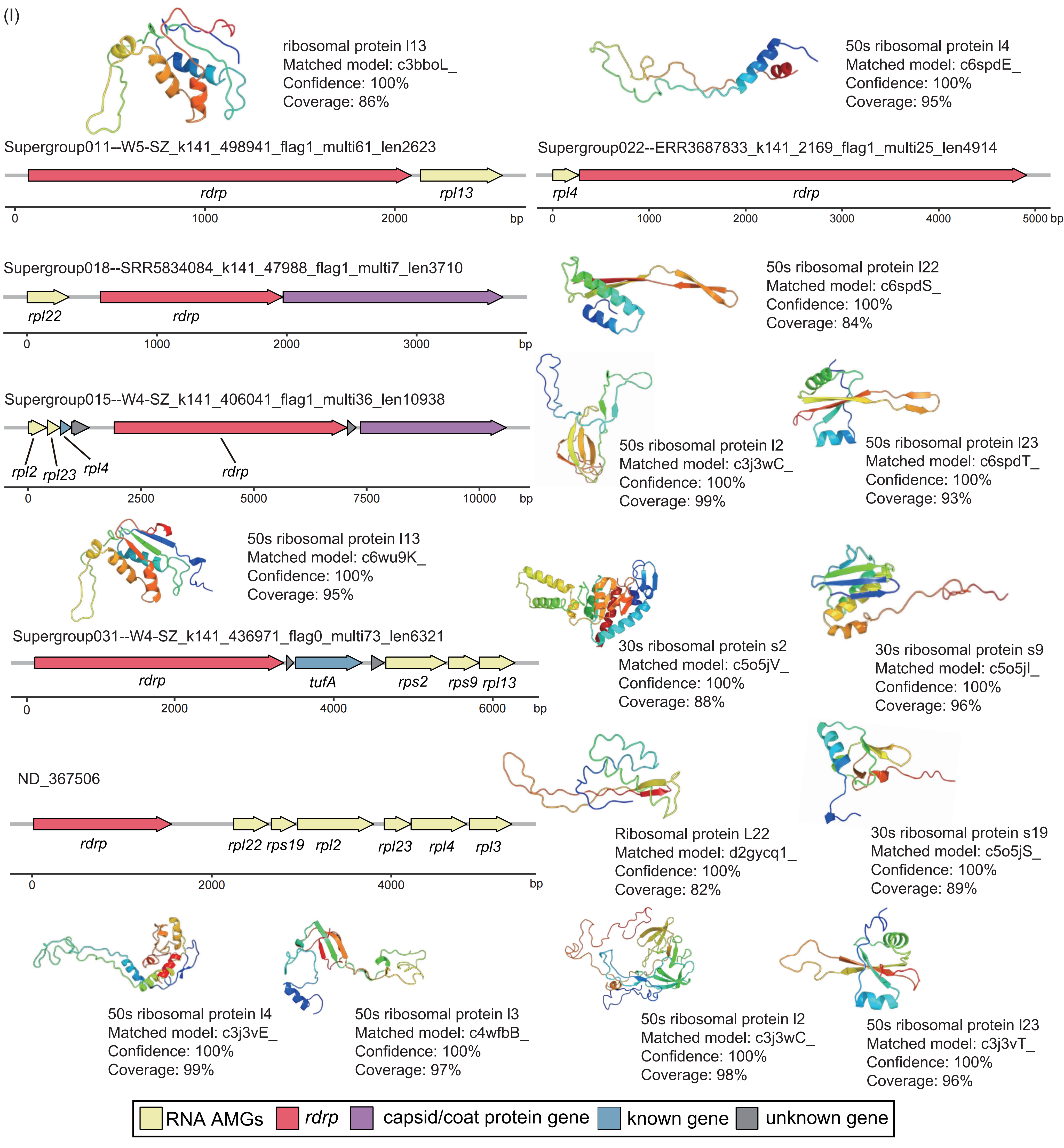


**Figure S9 Genome architecture of RNA vContig encoding a ribosomal protein gene and reference protein model.** (I) Genome architecture of RNA vContig encoding *rpl13, rpl4, rpl22, rpl2, rpl23, rps2, rps9, rps19* and *rpl3* gene and reference protein model for RPL13, RPL4, RPL22, RPL2, RPL23, RPS2, RPS9, RPS19 and RPL3.


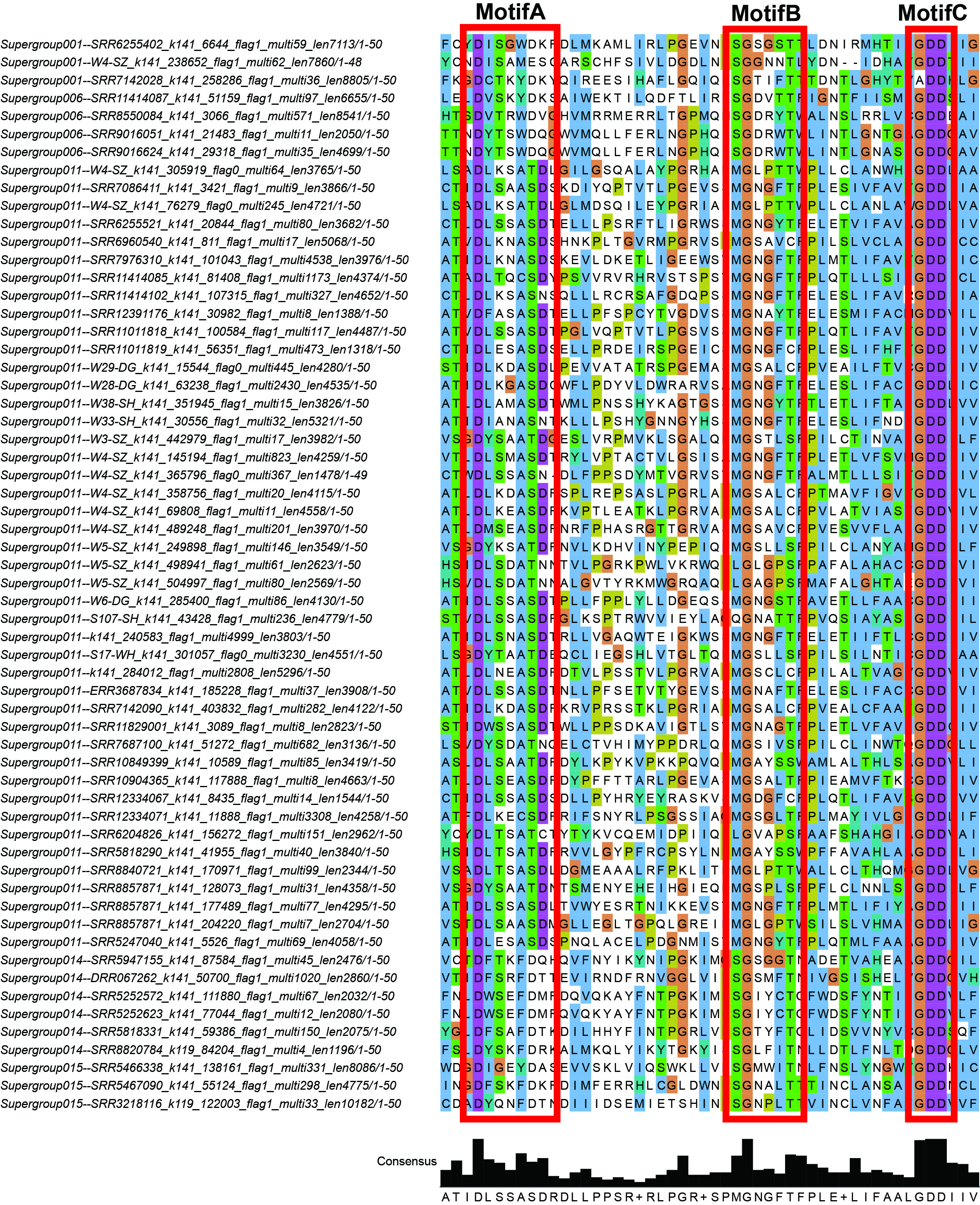


**Figure S10 RdRP sequence with three highly complete conserved sequence motifs (A, B, and C).**


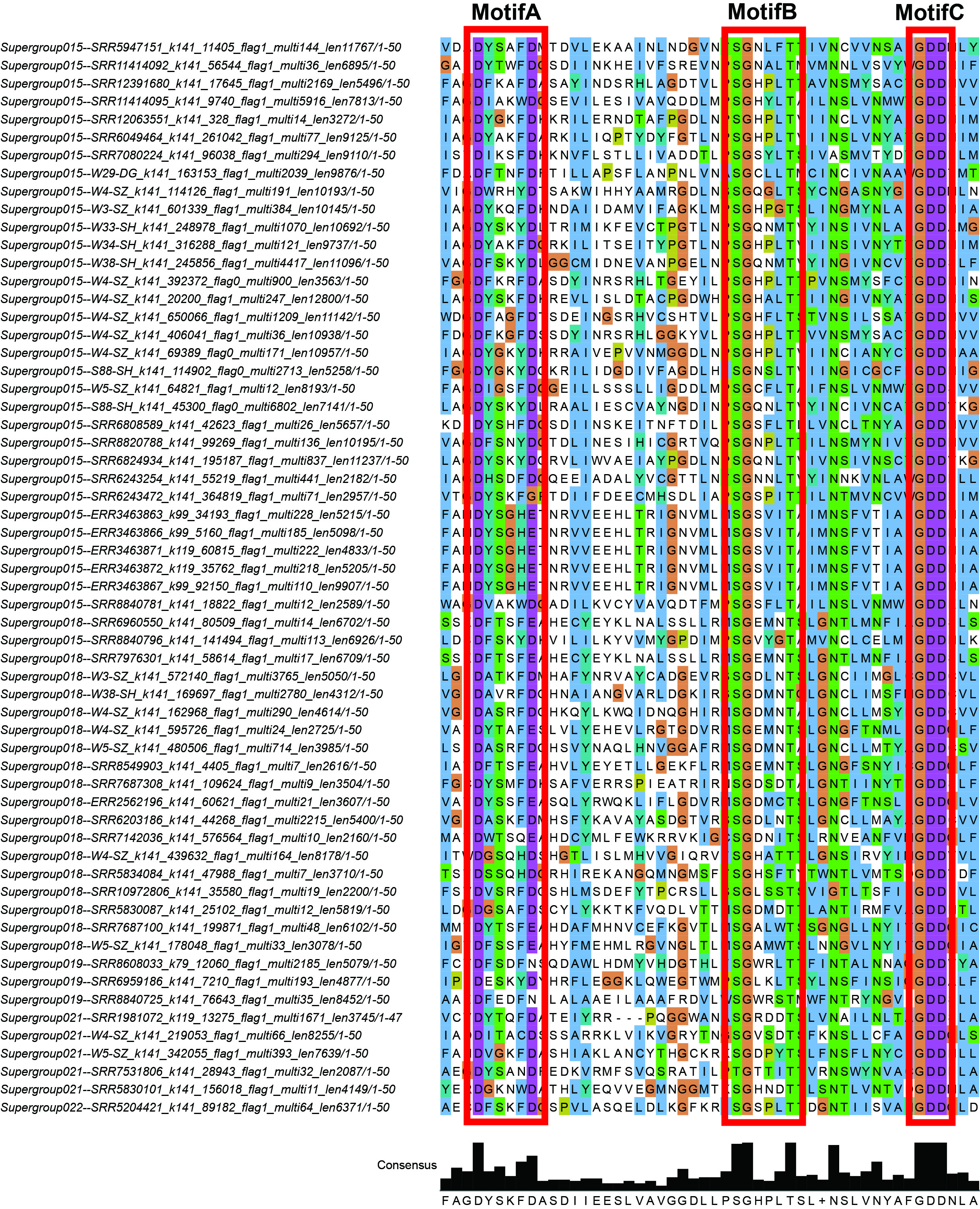


**Figure S10 RdRP sequence with three highly complete conserved sequence motifs (A, B, and C).**


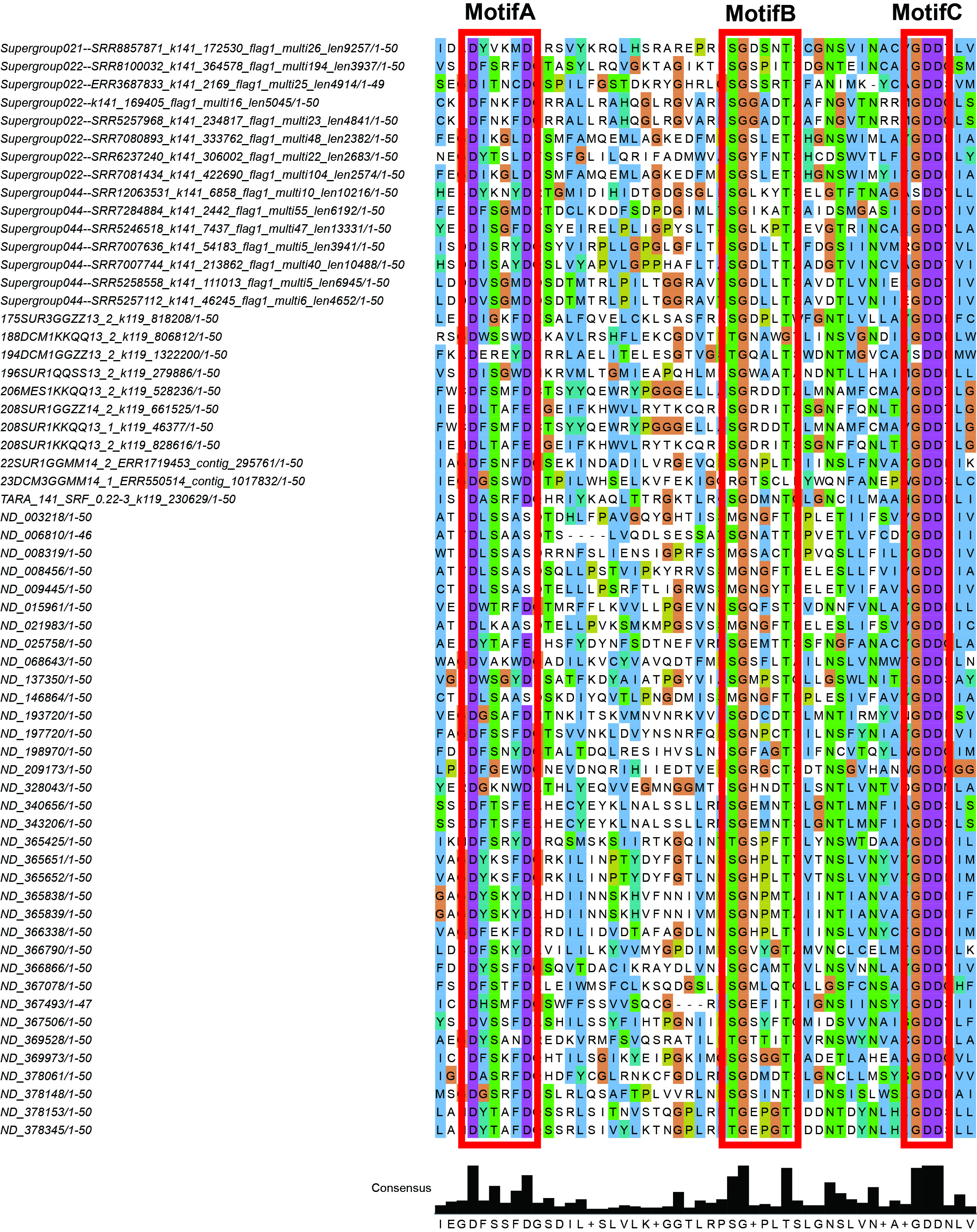


**Figure S10 RdRP sequence with three highly complete conserved sequence motifs (A, B, and C).**
